# Wearable All‐Fabric Hybrid Energy Harvester to Simultaneously Harvest Radiofrequency and Triboelectric Energy

**DOI:** 10.1002/advs.202309050

**Published:** 2024-02-21

**Authors:** Zhenghao Kou, Chao Zhang, Buyun Yu, Hao Chen, Zhenguo Liu, Weibing Lu

**Affiliations:** ^1^ State Key Laboratory of Millimeter Waves School of Information Science and Engineering Southeast University Nanjing 210096 P. R. China; ^2^ Center for Flexible RF Technology Southeast University Nanjing 210096 P. R. China; ^3^ Purple Mountain Laboratories Nanjing 210096 P. R. China

**Keywords:** FCB‐SMT, hybrid energy harvester, power management, rectenna, triboelectric nanogenerator

## Abstract

Distributed micro‐energy harvesting devices offer the flexibility, sustainability, and multi‐scenario applicability that will be critical to wearable electronic products in the Internet of Things. The radiofrequency and triboelectric (RF‐TE) hybrid energy harvester (HEH) concept and prototype is presented for the first time, to simultaneously capture the energy from ambient electromagnetic waves and biological motions. The proposed hybrid energy harvesting system consists of a wearable rectenna, a triboelectric nanogenerator (TENG), and a power management circuit (PMC). Among them, the all‐fabric rectenna exhibits good impedance matching characteristics in the ISM frequency. The flexible TENG unit can generate a maximum power density of 0.024 µW cm^−2^. The designed multifunctional fabric‐based PMC can considerably enhance the controllability of harvested hybrid energy. Additionally, a normalizable fabric circuit board quasi surface mount technology (FCB‐SMT) is proposed to integrate all modules on the same fabric substrate in one step, making the entire system superior mechanical robustness. The proposed wearable fabric‐based RF‐TE hybrid energy harvester is capable of successfully driving consumer electronics (such as sensors, watches, etc.). It provides a new energy solution strategy for self‐powered wearable electronic devices and is anticipated to encourage the efficient utilization of renewable energy.

## Graphical abstract

A novel flexible and wearable hybrid energy harvesting strategy is developed. The system consists of a rectenna, a triboelectric nanogenerator (TENG), and a power management circuit (PMC). An effective fabric circuit board quasi surface mount technology (FCB‐SMT) is proposed for realizing the all‐fabric hybrid energy harvester. This research could benefit the development of self‐powered wearable devices.

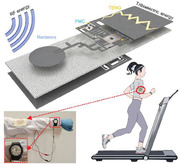

## 1 Introduction

The continual rise of wearable smart devices has filled people with hope for future technological living, and the Internet of Things age appears to be within approach.^[^ ^1^, ^2^, ^3^, ^4^, ^5^, ^6^ ^]^ Wearable electronic devices need to overcome the challenges of operating sustainably and stability. Accordingly, various types of wearable energy harvesters have emerged to minimize the reliance of distributed electronics on batteries. Solar cells, radio frequency (RF) rectennas, triboelectric nanogenerators (TENG), and thermoelectric generators (TNG) are the energy harvesters that are widely adopted.^[^ ^7^, ^8^, ^9^, ^10^, ^11^, ^12^, ^13^, ^14^, ^15^, ^16^, ^17^ ^]^ In practical application scenarios, it could be difficult to avoid the limitations of an insufficient or intermittent supply of a single energy source (such as sunlight, intermittent sports, etc.). Energy harvesting effectiveness can be significantly increased by developing the cooperative strategy of multiple energy sources, such as solar‐RF hybrid energy harvesters, solar‐bioenergy hybrid energy harvesters, etc.^[^ ^18^, ^19^, ^20^, ^21^, ^22^, ^23^, ^24^, ^25^, ^26^, ^27^, ^28^, ^29^, ^30^, ^31^ ^]^ The environmental‐biological hybrid energy harvesters with complementary characteristics of energy sources could ensure a more dependable energy supply for wearable electronic devices, because it may reduce the restrictions of energy sources. As a consequence, the novel environmental‐biological hybrid energy harvesting strategy is anticipated to improve the application potential of wearable devices.

The coverage of radio waves has expanded along with the increasing demand for wireless communications, making it easier to gather RF electromagnetic waves.^[^ ^32^ ^]^ In contrast to other forms of environmental energy, RF energy is less reliant on the natural environment and can be precisely artificially regulated in terms of a specific frequency, coverage area, and power intensity.^[^ ^33^, ^34^, ^35^ ^]^ At the same time, in the field of bioenergy harvesting, TENG has been favored by scholars thanks to its simple structure and a wide variety of material options. Wearable TENGs have been proven to be efficient at harvesting biomechanical energy.^[^ ^36^, ^37^, ^38^, ^39^, ^40^, ^41^, ^42^, ^43^ ^]^ Given this, we propose the radiofrequency and triboelectric (RF‐TE) hybrid energy harvesting strategy that may shine in the coming Internet of Things age.

However, the following challenges are required to be overcome to achieve the wearable fabric‐based RF‐TE hybrid energy harvester. First, the power management circuit (PMC) based on fabric materials should be prepared efficiently and reliably. The majority of reported power management components of fabric energy harvesters are rigid printed circuit boards (PCBs) disguised as garment buttons or decorations. PMC will have more elements and complex traces as its function continues to be enriched, and it will become harder to miniaturize and disguise. Thus, the largest obstacle to the full‐fabric realization of the energy harvesting system is the power management component. Second, the output characteristics of the harvested RF energy and triboelectric (TE) energy are vastly different. The RF energy captured by the antenna is a high‐frequency (GHz level) AC signal, whilst the TENG converts mechanical energy into low‐frequency (<10 Hz) AC output. Consumer electronics cannot be powered directly by either type of electrical energy. Hence, the design of matching power management is crucial.

In response to the above‐mentioned various challenges and needs, we propose an instructive and effective fabric circuit board quasi surface mount technology (FCB‐SMT) processing method. The compatibility of FCB‐SMT with rigid PCB‐SMT shows its potential for industrialization. By utilizing the FCB‐SMT procedure on the fabric substrate, the designed RF‐TE hybrid energy harvester achieves the integrated processing and assembly of the entire structure in one step. In addition, the designed PMC incorporates maximum power point tracking (MPPT), charge protection, and undervoltage lockout (UVLO) capabilities, allowing it to efficiently convert and store renewable energy sources with varying output characteristics. Finally, it is demonstrated that the designed hybrid energy harvesting system can efficiently harvest RF and TE energy, and the energy gathered can serve as renewable energy sources for various kinds of consumer electronics products (such as temperature sensors, watches, etc.).

## 2 Results and Discussion

### 2.1 Layout of the Hybrid Radiofrequency and Triboelectric (RF‐TE) Energy Harvester

**Figure 1:**
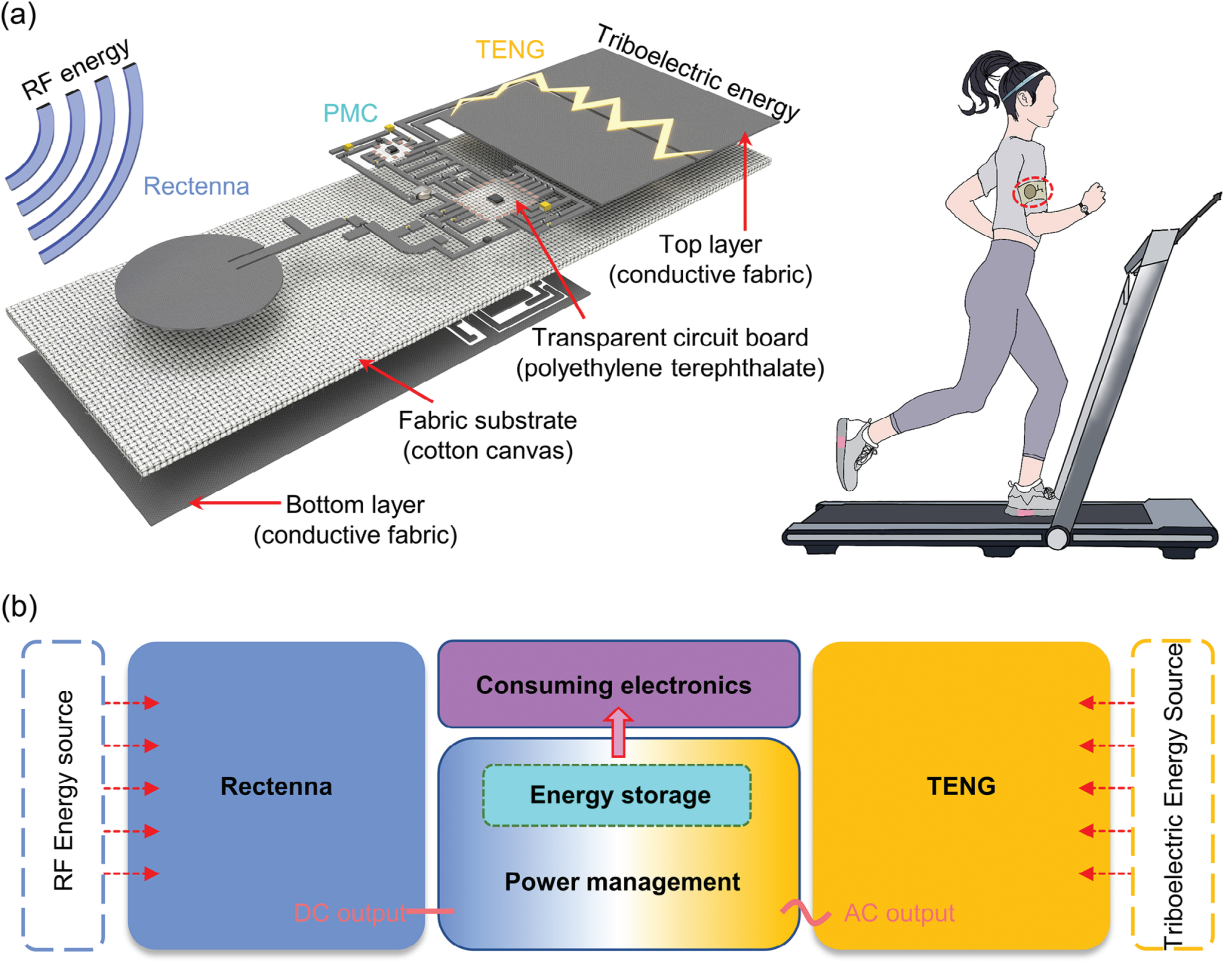
Schematic illustration of the RF‐TE HEH. a) Schematic diagram and scenario construction of radiofrequency and triboelectric (RF‐TE) hybrid energy harvester (HEH). b) System‐level schematic representation of energy transfer and conversion in the RF‐TE HEH operating engineering.

The fabric‐based rectenna, TENG, and PMC comprise the proposed all‐fabric hybrid energy harvester, which is illustrated in **Figure** **1**a. The corresponding operating mechanism of the hybrid energy harvesting system is depicted in Figure 1b. The antenna captures the RF electromagnetic waves from the surrounding environment, which is then rectified by the RF rectifying circuit into a low‐voltage DC output. The energy is subsequently transferred to an energy storage device, such as a supercapacitor or lithium battery, employing the PMC. The TENG is used to gather human kinetic energy and convert it into low‐frequency AC output, which is then transmitted to the same energy storage component through the PMC. A stable DC output with programmable voltage values could be produced by regulating the energy in the storage device for supplying various consumer electronics at the back end. The effective power management module of the hybrid energy harvesting system enables it to simultaneously harvest RF energy from the ambient environment and TE energy released by human body movement. Our design presents an innovative electronic prototype and paradigm that complements hybrid energy harvesting techniques and provides a fresh perspective on the challenges associated with the energy supply of wearable electronic devices.

Photo images of each module of the fabricated hybrid energy harvested are exhibited in Figure S1 (Supporting Information). The fabric of cotton canvas is chosen as the substrate of the entire hybrid energy harvesting system, which is widespread in daily life. It not only has good breathability but also can be freely bent or twisted to satisfy the wearable criteria. The canvas has a thickness of 1.28 mm. According to the microwave resonant cavity assessment, which is depicted in Figure S2 (Supporting Information), the relative dielectric constant (*ε*_r_) of cotton canvas is 1.67 and the dielectric loss tangent angle is 0.013. Additionally, commercial conductive fabric (bought from Taobao, an online shipping platform in China) is used to realize the metallic structure of the entire system, which includes the patch antenna, microstrip circuit, TENG, and PMC. The tungsten‐copper‐nickel alloy and polyester fiber are the essential ingredients of the conductive fabric, which not only has a high conductivity but also has the inherent benefits of fabrics, including excellent wearing comfort. The conductive fabric has a thickness of 0.09 mm. Furthermore, the conductive fabric square resistance of 4.409 × 10^−3^ Ω_□_ was measured using a four‐probe tester (model: Jingge M‐3), as shown in Figure S3 (Supporting Information). According to Equation (S2), Supporting Information, it has a conductivity of 2.52 × 10^6^ Sm^−1^, and the precise mathematical derivation is displayed in Note S1 (Supporting Information). The above‐measured data were used for the simulation design. The overall design of hybrid energy harvesting aims to reduce the size to a limit of 10 cm × 30 cm. This size is ideal to allow the entire energy harvesting system to be worn on an adult's upper arm, with the TENG (used to collect TE energy from arm movement during human activity) on the inside of the arm and the antenna (used to capture RF energy in the environment) on the outside of the arm. Obviously, it operates as effectively when attached to a flatter area of the body, like the chest.

However, a significant challenge is to ensure high‐precision processing and preparation while patterning flexible materials (especially fabrics) without harming the fragile substrate material. In response to this, we have specifically implemented an FCB‐SMT process to innovatively accomplish the integrated preparation of the full energy harvesting system in one step, and the specific processing flow will be elucidated in Section 2.5. Due to its excellent accuracy, efficiency, and batch‐processing capabilities, this approach shows the potential for commercialization. It should be noted that this FCB‐SMT is employed for processing all of the samples in the following chapters.

### 2.2 Fabric‐Based Rectenna for Harvesting Radio Frequency (RF) Energy

#### 2.2.1 Circular Patch Antenna

**Figure 2:**
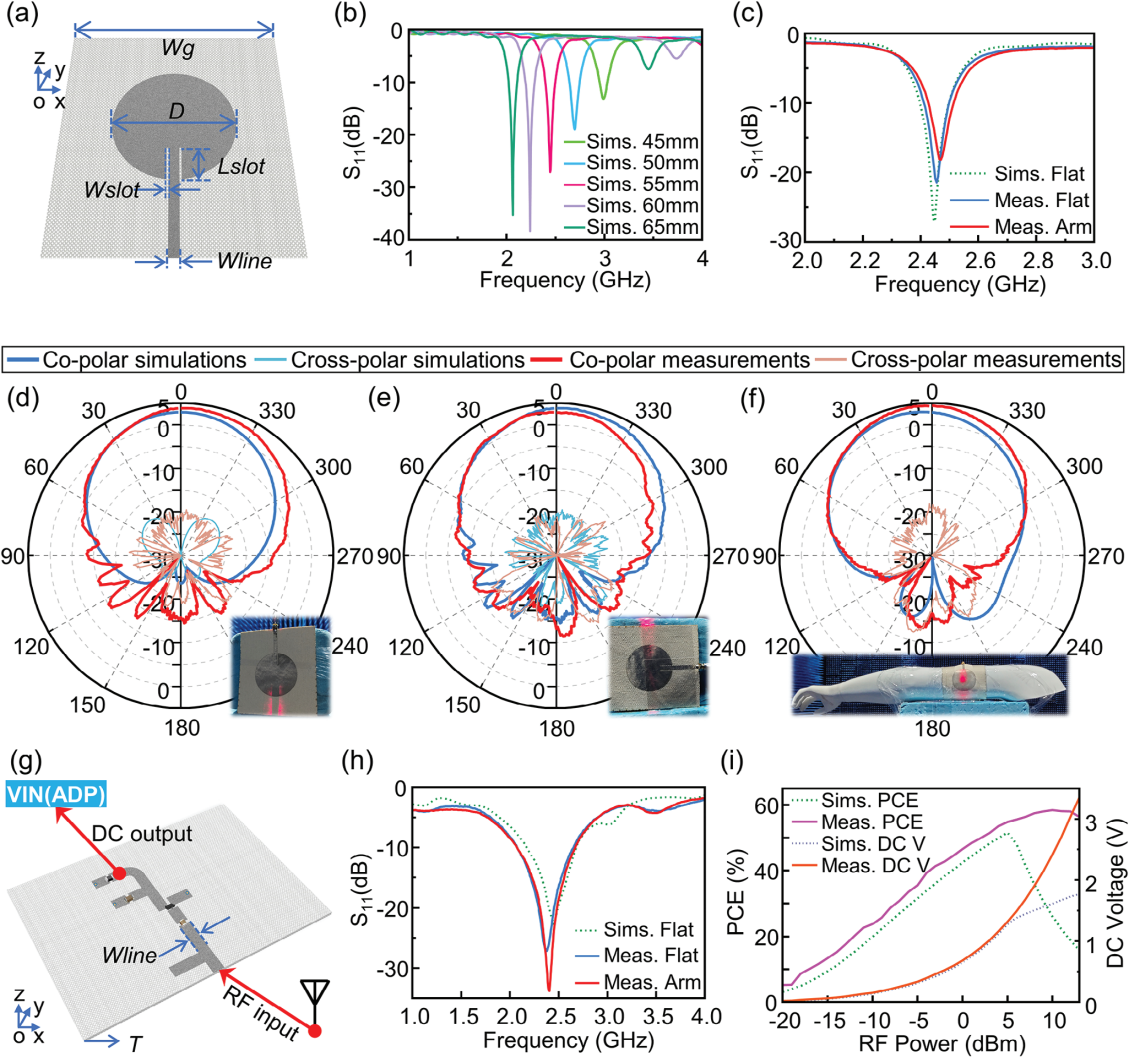
Characterization of the fabric‐based rectenna's performance. a) Schematic diagram showing the structure of the all‐fabric antenna. b) Simulated return loss of different diameters. c) Measured return loss of the fabricated antenna when it is flat and attached on a human arm. Measured and simulated radiation patterns of the fabricated antenna at 2.45 GHz: d) *xoz* plane when flat; e) *yoz* plane when flat; f) *xoz* plane bending on a human arm model. g) Schematic diagram showing the structure of the fabric‐based rectifying circuit. h) Simulated and measured return loss of the fabricated antenna when it is flat and attached on a human arm. i) Simulated and measured DC output and PCE of the fabricated rectifying circuit from varying input power.

The receiving antenna of the hybrid energy harvesting system will be worn on the body. To decrease the specific absorption rate (SAR) value and the effect of human body loading on the operation of the antenna, it requires minimal back radiation. To achieve this purpose, we developed an all‐fabric flexible wearable antenna capable of harvesting RF energy in the 2.45 GHz WiFi band. The proposed antenna has the advantages of high gain, low SAR value, excellent ruggedness, and compact structure. **Figure** **2**a depicts the antenna structure's schematic diagram.

In accordance with the simulation results in Figure 2b, diameter *D* of the circular patch has a significant impact on the resonant frequency of the antenna. To determine the appropriate size, it is required to first calculate *D* = 53 mm using the cavity model theory equation, Equation S3 (Supporting Information), and then simulate and optimize the patch diameter size using the CST STUDIO commercial full‐wave simulation software to set up the antenna resonance point close to 2.45 GHz, resulting in the final circular antenna diameter being *D* = 55 mm. In addition, a microstrip‐embedded feeding method was chosen, which not only enables direct integration of the antenna and the back‐end rectifying circuit but also makes it simple to test the performance of the antenna. The width of the microstrip feed line was set to 4.4 mm as displayed in Note S3 (Supporting Information), so that the characteristic impedance of the microstrip line is ≈50 Ω to minimize the error caused by the mismatch between the microstrip line and the port of the test instrument. Figure S4a (Supporting Information) depicts the simulation result of the antenna surface current distribution at 2.45 GHz, which is the TM_11_ mode current distribution of the circular patch antenna and is consistent with cavity model theory.^[^ ^44^, ^45^ ^]^ To determine if the designed antenna complies with the SAR value restriction, the SAR value of the antenna was also simulated and estimated. The results are shown in Figure S5 (Supporting Information). As can be observed, the peak SAR (1 g) is substantially lower than the 2 Wkg^−1^ international standard limit at 0.076 Wkg^−1^.^[^ ^46^, ^47^ ^]^

The off‐body and on‐body testing of the manufactured sample antenna was carried out separately with a vector network analyzer (model: Keysight N5227B) in order to experiment return loss (*S*_11_) parameter of the antenna designed by this study, as shown in Figures S6,  S7 (Supporting Information). The results are given in Figure 2c. The result of the off‐body tests aligned with that of the simulation. The center frequency of the antenna is 2.45 GHz, and the −10 dB impedance matching bandwidth is 2.41–2.5 GHz. It shows that the flexible antenna can effectively receive electromagnetic waves in the ISM band. The sample antenna can still maintain the −10 dB impedance match between 2.43 and 2.51 GHz although its center frequency shifts by 20 MHz when it is worn on the human arm. These results demonstrate that even after being bent and attached to a human body, the developed antenna still demonstrates exceptional robustness. This is due to the fact that, as shown in Figure S4b (Supporting Information), the current distribution of the circular patch antenna's primary mode of operation (i.e., the TM_11_ mode) mainly flows in the *oy* direction, and when it is bent around the *x*‐axis, the current distribution will not significantly change, keeping the mode of operation constant.

Another crucial indicator is the radiation pattern of the antenna. The antenna sample was placed in a microwave anechoic chamber for testing its radiation characteristics, and the test environment is illustrated in Figure S8 (Supporting Information). Initially, the realized gain pattern of the designed flexible fabric antenna operating at 2.45 GHz was tested when placed flat. The test results and corresponding simulation results of the *xoz* plane are shown in Figure 2d, while those of the *yoz* plane are presented in Figure 2e. The actual measurement results reveal that the designed all‐fabric antenna can achieve a maximum gain of 4.4 dBi when in the flat configuration, which is consistent with the simulation results. However, testing the radiation characteristics directly when the antenna is worn on the human body is challenging because the Device Under Test (DUT) must be mounted on a rotating stage in the microwave anechoic chamber. As an alternative, the antenna sample was strapped to the upper arm of a human body model to test its radiation characteristics in the bent *xoz* plane. The obtained test results and corresponding simulation results are shown in Figure 2f. The radiation pattern of the antenna in the curved case is not significantly different from that in the flat case, maintaining the maximum realized gain of 4 dBi. This is consistent with the mode analysis results of the antenna based on cavity model theory mentioned earlier.

The previously stated research results demonstrate that the designed antenna can dependably harvest RF energy of the 2.45 GHz WiFi band in the ambient space, whether it is worn on flat areas of the body like the chest, back, or other parts with larger curvature like the arms or legs.

#### 2.2.2 RF Rectifying Circuit

The design of the RF‐DC rectifying circuit is essential to the proposed wearable hybrid energy harvesting system. First, the rectifying circuit needs to be flexible to ensure optimal comfort while wearing. Second, the energy conversion efficiency should be maximized under the circumstances of electromagnetic wave incidence in the dynamic power range. Finally, processing accuracy should be enhanced because both the length and width of the microstrip line can have a substantial impact on the matching network of the rectifier.

Here, a wearable, high‐efficiency RF‐DC rectifying circuit made entirely of fabric is developed. The schematic diagram of the rectifying circuit on the fabric substrate is shown in Figure 2g, and the corresponding structural dimensions are listed in Figure S9 and Table S1 (Supporting Information). The RF‐DC energy conversion circuit and the receiving antenna are arranged in the same plane, which means they both face outward from the human body and share a common ground. This helps to make it easier to lay out the hybrid energy harvesting system overall. To facilitate integration and eliminate impedance mismatch, all microstrip lines are 4.4 mm wide, which is the same as the feeding line width of the fabric antenna. In this design, a full‐wave rectifier is implemented using a first‐order Greinacher‐type topology due to the increased output voltage facilitated by this structure. The principle of operation is depicted in Figure S10 (Supporting Information). In the rectifying circuit, additional components, such as two Schottky diodes (SMS7630) and two capacitors (100 pF) are installed. The diode in the RF rectifying circuit requires a stronger high‐frequency performance in contrast to the type used for back‐end rectification of TENG with low‐frequency AC output characteristics, for which the SMS7630 is a preferable option. Because the diode's input impedance is nonlinear, the rectifying circuit was designed and simulated by utilizing the harmonic balancing control module in the commercial software ADS employing the SPICE parameters for the SMS7630 diode that are provided on the official datasheet.

The off‐body return loss measurement was performed first to experimentally validate the impedance matching characteristics of the developed fabric‐based rectifying circuit. The rectifying circuit sample was connected to a vector network analyzer as indicated in Figure S11 (Supporting Information), and the S‐parameter results were obtained. Figure 2h displays the test‐derived *S*_11_ results along with the corresponding simulation results, and it is clear that the simulation results are aligned with the measured values. The rectifying circuit can achieve impedance matching of <−10 dB from 2.15 to 2.66 GHz, which completely covers the resonant frequency band of the front‐end receiving antenna. Furthermore, the range of frequencies between 2.34 and 2.49 GHz where *S*_11_ is <−20 dB offers the most ideal impedance matching. Additionally, when the fabric‐based rectifying circuit was bent and attached to the human body, its S‐parameters were investigated, as shown in Figure S12 (Supporting Information), and the results were also depicted in Figure 2h. The rectifying circuit can still perform stable impedance matching characteristics even when tested with human body loading by comparing the results of the off‐body and on‐body evaluations. This indicates the rectifying circuit can be easily cascaded and employed with the all‐fabric patch antenna for collecting RF energy.

The typical load is replaced by a 1 kΩ resistor that was soldered directly to the output in order to evaluate the rectifying circuit has the capacity to drive electronic devices as well as the related RF‐to‐DC power conversion efficiency (PCE). A portable RF source (model: SG‐3000PRO) with a maximum RF power of 13 dB mW was connected to the input of the rectifying circuit to imitate the collected RF energy. The output voltage of the resistive load was monitored under various incidence powers, and the recorded data results were displayed in Figure 2i. With a 13 dB mW RF power input, the maximum 3.35 V DC voltage output was achievable at the 2.45 GHz frequency point. Under 10 dB mW RF power input, the maximum RF‐to‐DC PCE reached a remarkable 58%. The RF‐to‐DC conversion efficiency is calculated by the following equation, $\eta_{RF-to-DC}=P_{RFload}/P_{RF}=V_{out}^{2}/\left(R_{Load}P_{RF}\right)$, where *P_RF_* is the output power of the RF energy source, and *V*_ou_ *_t_* is the voltage level at the resistive load. The actual DC voltage output of the flexible rectifying circuit and its corresponding efficiency will be a little greater since there will be some electromagnetic losses between the coaxial cable and the port connector.

The aforementioned results convincingly show that the proposed fabric‐based rectifying circuit can effectively convert RF energy into DC output. It should be specifically noted that we separated the RF rectifying circuit from the power management section, which not only made it easier to co‐design the circuit with the antenna but also increased the generalizability of the PMC. The PMC stated in Section 2.4 can thus be expanded to other appropriate applications. An example would be a thermoelectric‐triboelectric hybrid energy harvester.

### 2.3 Wearable Triboelectric Nanogenerator (TENG) for Harvesting Biomechanical Energy

In addition to using rectennas to harvest RF energy from the environment, a flexible fabric TENG is produced in this section to gather biomechanical energy and transform it into electricity, thereby enhancing the sustainability of wearable electronic devices.

After Academician Zhonglin Wang's initial proposal of TENG in 2012, extensive research by academics led to the formation of a largely finished theoretical and experimental system.^[^ ^48^, ^49^, ^50^, ^51^ ^]^ One of the four classical modes of TENG is the sliding freestanding triboelectric‐layer (F‐TENG) mode, which comprises a freestanding triboelectric layer and two electrodes in another layer. Not only are the two electrodes not always on the same plane, but the freestanding triboelectric layer also has a high degree of spatial freedom. As long as the conductive layer material and the triboelectric layer material have different triboelectric polarities, a potential difference can easily be formed between the two electrodes when the triboelectric layer reciprocates, leading to the reciprocation of electrons in the external circuit. TENG thus has a wide range of tolerance for material choice.

**Figure 3:**
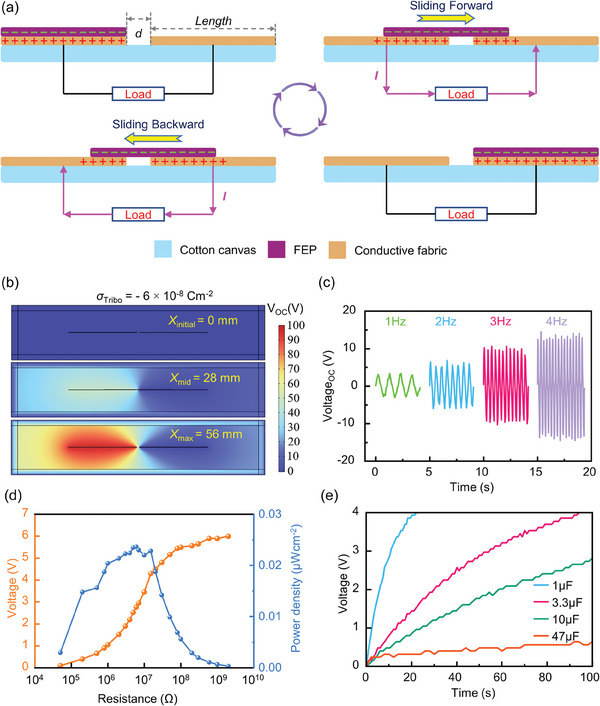
Working principle and output characteristics of the all‐fabric TENG. a) Schematic diagram of the working principle of the all‐fabric F‐TENG in contact sliding mode. b) The simulated potential distributions at three typical sliding displacements (initial, middle, and maximum). c) Electrical output (V_OC_) of the all‐fabric TENG under different sliding frequencies (1–4 Hz). d) Dependence of the TENG output voltage and power density on different load resistances. e) Charging different commercial capacitors by the fabric‐based TENG.

A sliding F‐TENG with good performance is developed to capture human kinetic energy while preserving the possibility of TENG integration with clothing. As illustrated in **Figure** **3**a, two 54 mm × 100 mm (Length × Depth) rectangular conductive fabrics are positioned adjacent to one another at a spacing of 2 mm (*d*) on a 110 mm × 100 mm rectangular canvas substrate. The conductive fabric employed here is the same as the above‐mentioned commercial conductive fabric, which facilitates the integrated processing of the metallic structure of all modules. The conductive fabric serves as both the counter triboelectric material and two electrodes. Commercial fluorinated propylene (FEP) film is used as the freestanding triboelectric layer to boost electrification between the two layers. The theoretical basis of F‐TENG is contact electrification and electrostatic induction. The four sequential steps of the entire reciprocal movement, as depicted in Figure 3a, can be used to explain the specific functioning mechanism. In the initial condition, neither the conducting electrode nor the dielectric FEP exhibits any net charge accumulation. According to the contact electrification effect, some negative charges are induced on the surface of the FEP when it comes into contact with the left electrode, and an equivalent quantity of positive charges are induced on the left electrode's surface. There is no current production at this time since the TENG system as a whole is in electrostatic equilibrium. Once the FEP film slides towards the right electrode, part of the positive charges on the surface of the left electrode loses the bondage of the negative charges on the surface of the FEP film and flows from the left electrode to the right electrode through the load in the outer circuit driven by the induced triboelectric potential. The FEP film is gradually split from the left electrode until the maximum separation state raises the triboelectric potential to its maximum value. The right electrode receives all positive charges through the external circuit when the FEP film overlaps it. The first half of the electricity generation cycle is currently completed. The FEP film then moves in the opposite direction from the right electrode to the left electrode until it reaches the overlapping position of the left electrode, driving positive charges back to the left electrode and causing a reverse current to flow via the external circuit. This is the second half cycle of the electricity generation process. According to this, FEP film reciprocates between the symmetrical electrodes, which will generate continuous alternating current in the external circuit.

The distribution of generated triboelectric potential can be calculated with the finite element simulation. The highest open circuit voltage between the two electrodes is positively associated with the amount of tribo‐charge density, as shown in Figure S13a (Supporting Information), which is readily extrapolated from the simulation results. In other words, the greater the charge density applied to the lower surface of the FEP film, the higher the open‐circuit voltage. Figure 3b shows the simulation outcomes of the generated triboelectric potential distribution for three typical sliding distances (*x*_initial_, *x*_mid_, and *x*_max_) when the tribo‐charge density (*σ*_Tribo_) on the lower surface of the FEP film is assumed to be −6 × 10^−8^ Cm^−2^. F‐TENG can create a maximum induced triboelectric potential of 96 V when the FEP film slides from the left electrode to the right until it overlaps with the right electrode. The set tribo‐charge density is also closely correlated with the quantity of charge transferred under short circuit conditions. The corresponding maximum transferred charge density (Δσ_SC_) under the same tribo‐charge density is 32.1 nCm^−2^, as shown in Figure S13b (Supporting Information).

Similarly, a TENG sample with an all‐fabric structure was processed to assess its electrical output performance and power density. The freestanding triboelectric layer (FEP film) was moved back and forth between the two electrodes by the linear motor, which has a maximum working frequency of 4 Hz. The two electrodes (conductive fabric) were connected to the test port of the oscilloscope through wires. At 4 Hz, the open‐circuit voltage reached a maximum of 14.5 V, as shown in Figure 3c. However, the walking frequency of adults is 1 Hz–2 Hz. The open circuit voltage that TENG can achieve at 2 Hz, which is closer to the actual application scenario, was 6.9 V. By connecting two electrodes to the ends of the multimeter, the short‐circuit current of TENG was measured, and the maximum short‐circuit current was 8 nA. It becomes difficult for us to measure the quantity of charge transmitted between two electrodes of TENG throughout an entire cycle due to the limitations of experimental testing instruments. However, another procedure was used to assess the highest instantaneous power density of the planned TENG. The two electrodes of TENG were attached to the port of a self‐designed 11‐bit programmable resistor, as shown in Figure S14 (Supporting Information). An oscilloscope was used to monitor how the voltage values across the resistor change as the resistance value varies, and the results are presented in Figure 3d. According to experiments, when the resistance was 6 MΩ, the highest instantaneous power obtained is *P = U^2^/R*, more accurately, equivalent to the maximum instantaneous power density of TENG was 0.024 µW cm^−2^. The charging capacity of the all‐fabric TENG was also evaluated, that is, capacitors with various values (1 µF, 3.3 µF, 10 µF, 47 µF) were connected directly after the two electrodes of the TENG without utilizing a power management system. Figure 3e illustrates that although a 47 µF capacitor requires 100 s to charge to 0.64 V, a 1 µF capacitor only requires 21 s to charge to 4 V at 2 Hz. Furthermore, we systematically assessed the impact of variables such as applied force, contact area, contact materials, and durability on the performance of the proposed TENG, as depicted in Figure S15 (Supporting Information). The experimental results reveal that increasing the force or expanding the contact area significantly enhances the TENG's output performance. However, the current use of three freestanding layer triboelectric materials (PE, PET, and FEP) provides only marginal improvement to the TENG's performance. Notably, applying pressure with a 200 g weight, and utilizing FEP as the freestanding layer triboelectric material, allows the TENG to maintain a stable output even after 1000 cycles, showcasing the excellent durability of the proposed TENG.

The aforementioned findings show that the fabric‐based TENG can efficiently capture human motion energy, and may be used as a dependable energy source.

### 2.4 Fabric‐Based Power Management Circuit (PMC)

Although the front‐end RF rectenna has converted high‐frequency electrical waves into DC voltage output, there is still a significant impedance differential between the output end of the rectifying circuit and the TENG. The former has a kiloohm‐level internal impedance, as shown in Figure S16 (Supporting Information), while the other has a megaohm‐level internal resistance. Furthermore, the power density of rectennas and TENGs might differ greatly. Consequently, it is often challenging to efficiently collect energy in multiple output forms simultaneously.

**Figure 4:**
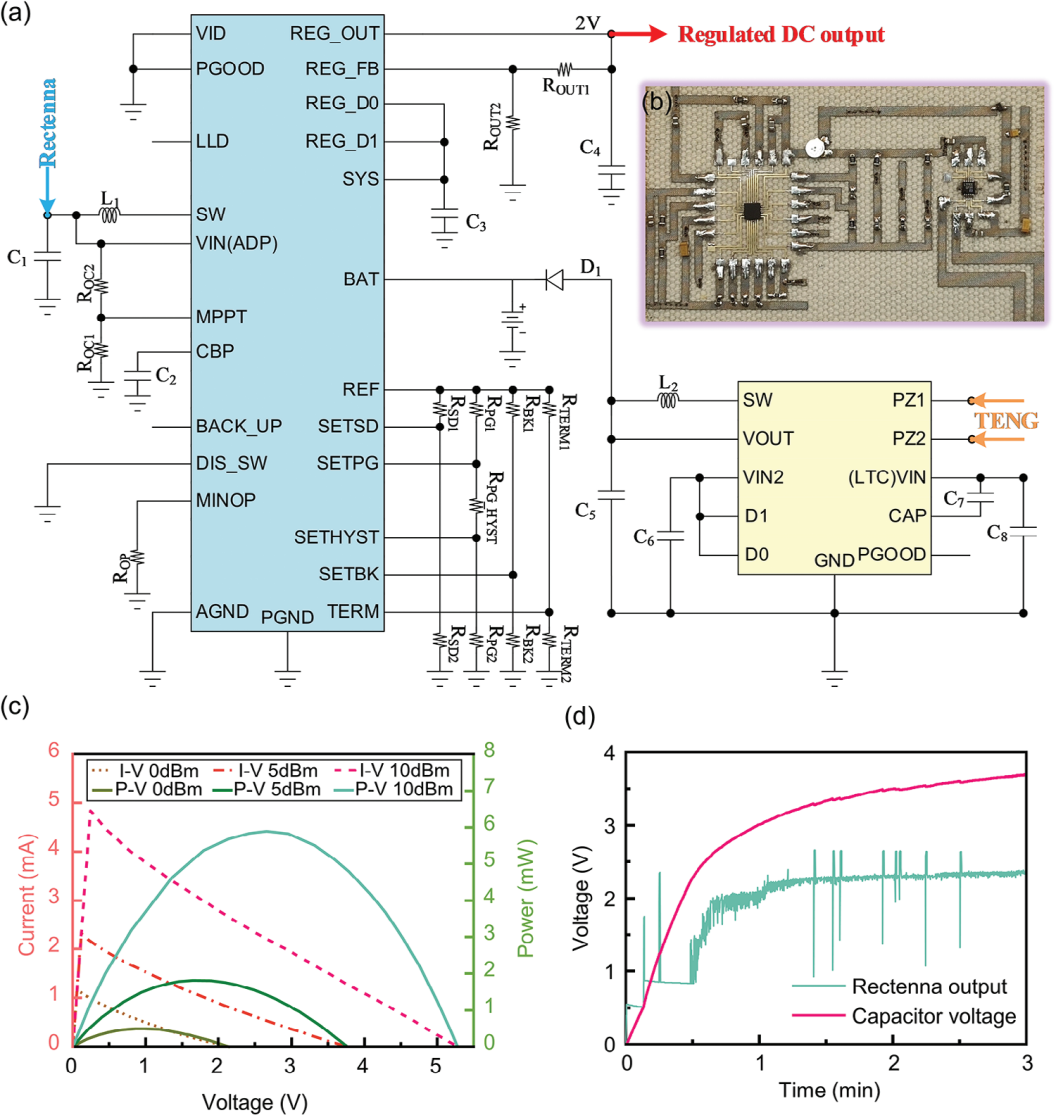
Schematic illustration of the fabric‐based PMC. a) Circuit diagram of the fabric‐based PMC module. b) Top view of the fabricated PMC. c) Current (left) and power (right) characteristics of the fabric‐based rectifying circuit for different input RF powers (0, 5, and 10 dB mW). d) Measured voltage waveforms at the VIN (ADP) and BAT pins of the fabric‐based PMC within the first 3 min.

To solve the problems mentioned above, a power management circuit (PMC) with maximum power point tracking (MPPT) function using open circuit voltage (OCV) proportional ratio method, undervoltage lockout (UVLO) function, and charge protection function is designed on the fabric substrate. The designed PMC can merge the energy obtained from the rectenna and the TENG into the same energy storage device (supercapacitor or lithium battery), enabling it to supply a sustained and reliable power source to the back end of consumer electronics. The fabric‐based PMC, which primarily comprises two MCU integrated chips (ADP5091 and LTC3588) and peripheral circuits, is presented in **Figure** **4**a,b to demonstrate its operation. The output of the rectenna is connected in series to the VIN pin of the ADP to collect RF energy, while the two electrodes of the TENG are connected to the PZ1 and PZ2 pins of the LTC to collect TE energy. Both obtained energies are then transferred to the energy storage element at the BAT pin of the ADP. The REG_OUT pin of ADP can offer a regulated voltage output to drive a variety of consumer electronics when the voltage of the energy storage element reaches the predetermined SETSD threshold. The MPPT function, UVLO function, and charge protection function of the PMC are described in detail below.

The MPPT function based on the OCV scaling factor algorithm has been widely employed in the field of solar and thermoelectric energy harvesting,^[^ ^52^, ^53^, ^54^ ^]^ where the typical MPPT ratio depends on the type of energy source. It is clear from the experimental data in Section 2.2.2 that various RF incident powers cause the voltage at the rectifying circuit output to vary significantly at the same frequency. Fortunately, even if the RF incoming power changes over a broad range, the voltage on the load corresponding to the rectifying circuit reaches the maximum power point, which is nearly constant in proportion to its OCV. The ADP chip takes a sample of the OCV at the terminal of the rectenna and stores it in the capacitor between the CBP pin and GND. According to the MPPT ratio programmed by the resistors (*R*_OC1_ and *R*_OC2_) on both sides of the MPPT pin, the DC–DC converter inside the chip changes the duty cycle of the internal switching tube control signal to change the input impedance of the VIN (ADP) port, making the PMC match the impedance of the rectifying circuit. As a result, the rectenna will be able to operate close to its maximum power point and gather as much energy as feasible under a variety of RF incident power intensities. The output terminal of the rectifying circuit is connected to the port of the self‐designed programmable resistor to determine the MPPT ratio of the RF rectifying circuit under three typical RF incident power conditions: 0 dB mW, 5 dB mW, and 10 dB mW. By adjusting the resistance value, the maximum power point of the rectifying circuit may be discovered. Figure 4c displays the *U*–*P* curve obtained from the experiment. Simple calculations show that the OCV to maximum power point voltage ratios for the three observed RF incident power circumstances were 0.42, 0.5, and 0.45, respectively, indicating that the MPPT ratio of the rectifying circuit was ≈0.5. In this design, the MPPT ratio was set to 0.5 in accordance with the findings of the previous tests. With reference to Equation (S13), Supporting Information, the corresponding resistances of *R*_OC1_ and *R*_OC2_ were utilized to program and set, which were 10 and 10 MΩ, respectively.

High efficiency power management is essential because of the inherent high voltage AC output characteristic of TENG. In this design, the integrated chip LTC3588, which has a UVLO capability with a wide hysteresis window, is used to effectively transfer the micro‐energy gathered by the TENG to a common energy storage element. When the TENG harvests energy, the output alternating current first flows through a low‐loss full‐wave rectifier bridge integrated between the PZ1 and PZ2 pins. The rectifier bridge can prevent energy loss caused by electrons flowing back to the TENG in addition to rectifying the AC output of TENG. After rectification, the electrical energy is pre‐stored in the input capacitor (*C*_8_) between the VIN(LTC) pin and GND. When the voltage across the input capacitor gradually reaches the UVLO threshold (typically 4.2 V), the buck converter inside the chip is activated and transfers some of the energy from the input capacitor to the output capacitor (*C*_5_) between the VOUT pin and GND. The buck converter will be switched off when the input capacitor voltage falls below the UVLO threshold, and it will not be turned back on until the input capacitor voltage reaches the UVLO threshold once more. Energy consumption during converter sleep is kept to a minimum by the extremely low quiescent current (450 nA typical) of UVLO. Electric energy will be delivered to the common energy storage element through the diode when the output capacitor (*C*_5_) progressively accumulates enough energy and generates a voltage differential with it. It should be noted that tantalum electrolytic capacitors, rather than ceramic capacitors, were used in both the input and output capacitors. Tantalum capacitors perform well in terms of power storage, high‐efficiency charge and discharge, and are particularly suited for micro‐energy storage and transfer because of their lower leakage current.

The function of charge protection of energy storage elements is crucial in the field of self‐powered wearable electronic devices to extend the service lifetime. The charging termination voltage of the energy storage element can be controlled by modifying the resistance values of *R*_TERM1_ and *R*_TERM2_ by Equation (S16), Supporting Information, effectively preventing the energy storage element from being destroyed by overcharging. The chosen resistors for the resistor divider in this design were 5.9 and 4.12 MΩ, respectively, as the set charging termination voltage was 3.6 V. The earlier mentioned programming control resistors have been chosen with relatively high resistance values to limit the quiescent current and minimize the energy consumption of the power management system. Other specific lumped element configurations are detailed in Table S2 (Supporting Information).

It is crucial to explicitly state that the entire PMC system operates without the need for external energy supply in this design. The energy necessary to keep the low‐power PMC operating originates exclusively from the harvested RF and TE energy. The developed power management system can also be extended to additional hybrid energy harvester applications with DC–AC output characteristics, such as solar‐triboelectric or thermoelectric–triboelectric, by altering the specific configuration.

### 2.5 Fabric Circuit Board Quasi Surface Mount Technology (FCB‐SMT)

New wearable electronic devices are progressively becoming more intelligent and lightweight in the Internet of Things age. Various RF devices and electronic circuits should also be designed and processed on the fabric substrate to better integrate with apparel. However, fabric materials cannot tolerate high temperatures and are prone to deformation or other drawbacks. This means that conventional processes, like PCB based on rigid substrates, are not immediately adequate for fabric substrates. Thus, there are numerous difficulties in achieving high precision and efficiency in the fabrication of RF devices and electronic circuits based on frail fabric materials.

**Figure 5:**
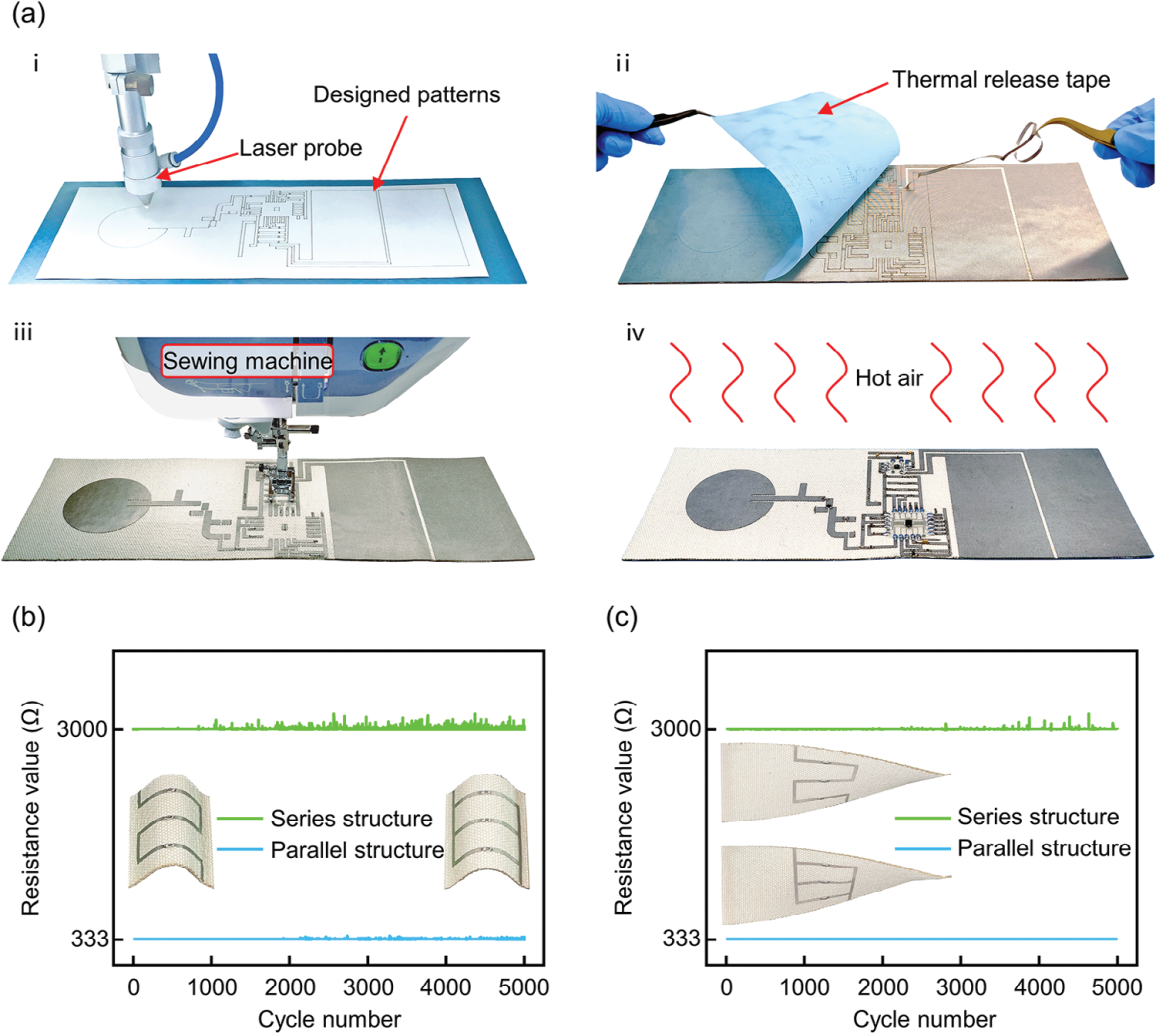
Summary of the fabrication of FCB‐SMT and fabricated modules. a) The processing flow of FCB‐SMT: i) metallic structure patterns cutting; ii) transfer and thermal releasing; iii) metalized vias stitching; iv) lumped components assembly. Results of fatigue tests performed on circuits in series and parallel configurations under (b) bending situations and (c) twisting situations.

The present study innovatively employs the FCB‐SMT technique to realize the preparation of the RF‐TE hybrid energy harvesting system on the fabric substrate first, overcoming the aforementioned difficulties. This technique has the advantages of high precision and high efficiency, retaining its potential for industrial mass production, and can realize the one‐step processing and molding of the entire energy harvesting system (including antenna, RF rectifying circuit, TENG, and PMC). As shown in **Figure** **5**a, the proposed FCB‐SMT process is primarily divided into four steps: metallic structure patterns cutting, transfer and thermal releasing, metalized vias stitching, and lumped components assembly. The first step is the patterned cutting of the metallic structure. Figure 5ai) at room temperature, adhere the non‐adhesive side of the commercially available conductive fabric (the opposite side is self‐adhesive) to the surface of the thermal release tape, and then cut it with a laser probe of the proper power. Patterned cutting enables the laser to penetrate through the conductive fabric with the least amount of thermal release tape penetration. The pattern of the metallic structure needs to be pre‐processed with mirror symmetry. During this step, only the conductive fabric attached to the thermal release tape is etched, ensuring that the fragile fabric material substrate remains unaffected. The second step is the transfer and thermal release of the conductive fabric. Figure 5aii) the adhesive side of the conductive fabric is attached to the fabric substrate, and the thermal release tape is heated at 130 °C by a heating device or an oven. The stickiness of thermal release tape will be greatly weakened, and it can be easily peeled off from the surface of the conductive fabric, and then the extra conductive fabric is peeled off to leave the desired metallic structure. The fabric material needs withstand a brief heating to 130 °C at this stage. The third step is the stitching of metalized vias. Figure 5aiii) following the completion of the above steps for the top and bottom layer metallic structure, utilize a sewing machine (model: Brother V5LE) to stitch or embroider at the locations where metalized vias are necessary. To ensure the strength of the stitching location, the upper thread and lower thread employ commercial silver‐coated conductive threads (bought from Taobao) and are stitched with the proper tension. The commercial conductive thread has a diameter of 0.17 mm, a silver content of more than 18%, and a resistance of 3.6 Ωcm^−1^, as shown in Figure S17 (Supporting Information). The fourth step is the lumped component assembly. Figure 5aiv) place the lumped components in their correct positions after applying an adequate quantity of low‐temperature solder paste (melting point of 138 °C) to the interconnection between the metallic structures and the components. We utilize an acrylic plate to cut the necessary holes at the solder joints to apply solder paste, much like the stencil used in PCB‐SMT, as shown in Figure S18 (Supporting Information). An oven or other heating device melts the solder paste, and after cooling, the components can be mounted. The lumped components (resistors, capacitors, inductors, diodes, etc.) are all packaged with SMD components, which can minimize their space requirements and thus ensure wearing comfort. During this stage, the fabric material needs endure a brief heating to 138 °C. The above is the entire process of the FCB‐SMT process. The fragile fabric material is heated to a maximum of 138 °C twice throughout the entire operation. Such a low temperature will not cause damage to the fabric material.

Additionally, a “pins expansion” strategy based on the ultra‐thin PET was used to enhance the assembly precision and operational stability of the MCU integrated chips. This entails designing the “pins expansion” circuit of the matching chip package on the PET substrate, as shown in Figure S19 (Supporting Information), and enlarging the pin to the same width as the conductive fabric trace (2 mm). The MCU chip on this PET circuit board should be pre‐soldered and take part in the aforementioned component mounting procedure as an equivalent “component assembly.” This method allows for better electrical connections between chip pins and fabric circuit traces without sacrificing the benefits of the lightweight and softness of the fabric substrate.

In order to confirm the stability of the electrical connection performance of circuits generated using FCB‐SMT, two fundamental types of fabric circuits were processed and performed fatigue tests in bending and torsion forms, as shown in Figures S20,  S21 (Supporting Information). Three 1000 Ω resistors were connected in parallel and series respectively. The trace width of 2 mm equals the power management circuit's trace width. The resistors were placed at the greatest bending curvature during the bending test. The push‐pull range was 2 cm, and the canvas substrate was 5 cm in length. The greatest torsion angle in the torsion test was 90°, and the fabric substrate length was 15 cm. Here, the circuit can still maintain a steady electrical connection despite bending or twisting after 5000 cycles, as shown in Figure 5b,c.

The proposed FCB‐SMT method features a standardized process and offers advantages in terms of low cost and large‐area preparation. Nevertheless, it cannot ensure the waterproofness of fabric‐based electronic systems. Thus, it can be applied to the rapid preparation of large‐scale fabric‐based electronic systems with low water resistance requirements.

### 2.6 Test of RF‐TE Hybrid Energy Harvesting System

RF‐TE hybrid energy harvesting system can power various wearable electronics by harvesting both RF energy and TE energy. The following tests were conducted to confirm the validity of this design.

**Figure 6:**
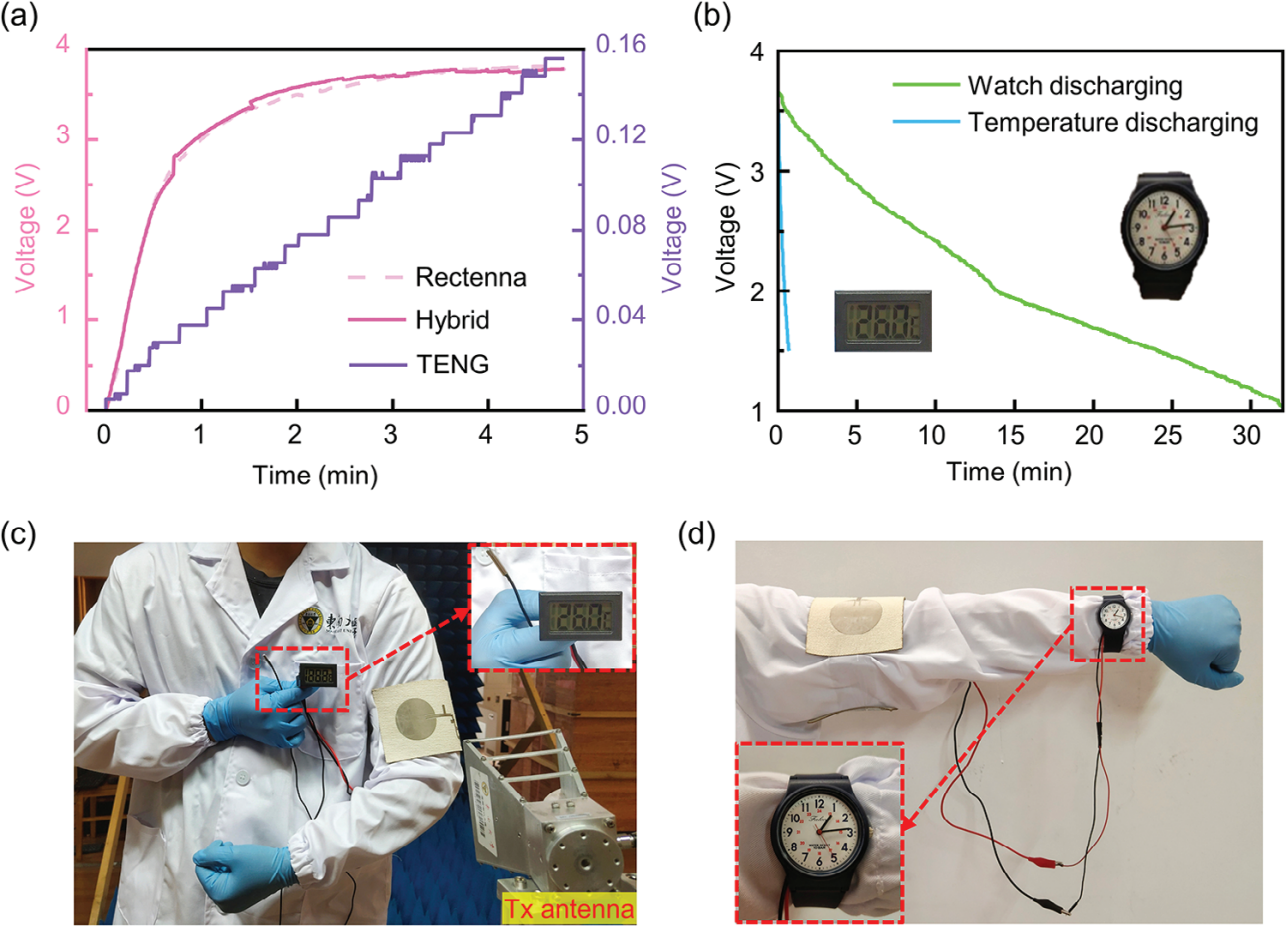
Demonstrations of the RF‐TE self‐powered system. a) Charging curves for a commercial 1000 µF supercapacitor through the fabric‐based PMC by RF rectenna, TENG, and both of which are operating simultaneously. b) Discharging curves of self‐powered commercial temperature sensor and watch. Photographs of a volunteer wearing the fabric‐based hybrid energy harvester to drive the (c) temperature sensor, and (d) watch.

First, the RF energy harvesting capabilities of the device were independently evaluated. The test setup is depicted in Figure S22 (Supporting Information). To imitate an RF energy source, a transmitting horn antenna situated 12 cm away from the fabric antenna was fed with 13 dB mW of RF power. The captured RF energy was then stored in a 1000 µF supercapacitor after rectification and power management. The results depicted in Figure 4d can be attained by simultaneously monitoring the voltage across the energy storage element and the fluctuation in voltage at the rectenna's output terminal (i.e., the VIN pin of ADP). The output waveform of the rectenna in the figure reveals that after the chip achieves a fast cold start‐up, it initiates the charging of the energy storage element at the BAT pin. Furthermore, the chip can dynamically detect the open circuit voltage and adjust the input voltage based on the set MPPT coefficient. During this phase, the boost regulator inside the chip operates in asynchronous mode. As the voltage of the energy storage element approaches the battery terminal discharging threshold programmed at the SETSD pin, the boost regulator inside the chip transitions to the synchronous working mode. Simultaneously, the MPPT control loop regulates the input voltage, continuously increasing it. The VIN pin is linked to the SW pin through an inductor, and the SW pin serves as the switching node of the boost regulator integrated inside the chip. The switching action causes substantial up and down spikes in the input voltage. Moreover, it can also be caused by the boost regulator briefly stopping when the open voltage sampling circuit dynamically senses the input voltage. Second, the device's capacity to capture TE energy was also examined separately. The artificial mechanical energy source was created by the reciprocating movement of a linear motor. Following power management, a 1000 µF supercapacitor was employed to store the captured TE energy. The voltage growth curve is not continuous, as depicted in **Figure** **6**a, but rather rises stepwise. This is due to the hysteresis of the PMC's UVLO function, and the capacitor *C*_8_ can only store the electricity that crosses the threshold before transmitting it to the back‐end energy storage element once. Third, the system's capacity for harvesting both RF and TE energy simultaneously was investigated. The identical 1000 µF supercapacitor stored all of the acquired energy. The findings are displayed in Figure 6a, and it indicates that hybrid energy harvesting had the fastest charging speed. The voltage across the energy storage capacitor stabilized at roughly 3.7 V after 3 min of charging and did not climb further. This corresponds to the preset 3.6 V charging cut‐off voltage. The charging protection mechanism prevents the energy storage element from being overcharged, extending the service life. The average charging power of the hybrid energy harvesting mode for 3 min under the experimental conditions in this paper amounts to 38 µW, and the maximum instantaneous charging power can reach 111 µW. The charging speed is applicable to some types of self‐sustaining low‐power IoT devices.

Finally, two specific self‐powered application scenarios for wearable devices were demonstrated as proof‐of‐concept. In Scenario 1, the fabric‐based HEH was bent and worn on the volunteer's arm. The energy collected can be utilized to power a commercial temperature sensor, which requires a voltage higher than 1.5 V for operation, as illustrated in Figure 6c. The voltage reduced from 3.65 V to 1.5 V after 42 s as indicated by the energy storage element's discharging curve, which can be seen in Figure 6b. Scenario 2 was a self‐powered demonstration of a commercial mechanical watch with a working voltage >1 V, as shown in Figure 6d; Video S1 (Supporting Information). The watch can continue running effectively for up to 32 min under the same circumstances.

## 3 Conclusion

In conclusion, we have successfully demonstrated a prototype of a novel wearable fabric‐based RF‐TE HEH that includes RF rectenna, TENG, and power management components. The all‐fabric metallic structure has an integrated layout and is compatible with an efficient and extendable FCB‐SMT process. The fabric‐based rectenna's architecture allows it to efficiently harvest ambient RF energy. The exhibited flexible TENG can efficiently gather (bio)kinetic energy. The integrated PMC is able to combine and regulate the different forms of energy that have been accumulated into a DC output. Finally, the hybrid energy harvesting system application scenario was successfully demonstrated as a source of power for some wearable devices (temperature sensors and watches). The fabric‐based hybrid energy harvesting system's operation also illustrates the entire cycle of energy harvesting, management, storage, and application.

The proposed all‐fabric RF‐TE hybrid energy harvesting system offers an innovative strategy for finding renewable energy sources for environmentally friendly self‐powered devices, which will motivate further study, system integration, and industrialization growth. One expectation is that the proposed fabric‐based RF‐TE hybrid energy harvesting system and FCB‐SMT method may hasten the development of wearable self‐powered consumer electronics for the Internet of Things.

## 4 Experimental Section

### Electromagnetic Characteristic Measurement of the Cotton Canvas

A split post dielectric resonator from QWED (model: F‐SPDR‐5.1) was utilized to measure the electromagnetic properties (relative dielectric constant and dielectric loss tangent angle) of the commercial cotton canvas. A vector network analyzer (model: Keysight N5227B) was used to first measure the resonant frequency and Q factor of the empty resonator. The pre‐prepared 5 cm × 5 cm canvas sample to be evaluated was subsequently inserted into the resonator cavity where it will cause the resonant frequency and Q factor to shift, as shown in Figure S2 (Supporting Information). The dielectric constant and dielectric loss of the canvas material were calculated by the difference of resonant frequency and Q factor, respectively.

### Simulation of All‐Fabric Antenna

CST STUDIO electromagnetic components analysis and design software were utilized to simulate and optimize the all‐fabric patch antenna. First, a microwave & RF project was created, and after choosing the planar antenna workflow the center frequency of the all‐fabric antenna was defined at 2.45 GHz. The three‐dimensional model was subsequently built employing the antenna dimensions estimated with cavity model theory. Then, the conductive fabric material (measured conductivity) was assigned to the patch and ground, while the canvas material (measured permittivity and loss tangent) was assigned to the dielectric layer. Following that, a macro tool was employed for setting up the wave port excitation at the end of the microstrip line. Finally, the antenna model was simulated using the time‐domain solver, and the results, such as the S‐parameters and the radiation pattern of the fabric antenna, can be obtained as displayed in Figure 2.

### Simulation of the Fabric‐Based RF Rectifying Circuit

The all‐fabric RF rectifying circuit was designed and simulated using the Advanced Design System (ADS) electronic design automation software. Firstly, the rectifying diode (SMS7630), which is not listed in the component library, needs to be modeled and packaged beforehand. The doubler voltage rectifying circuit of the Greinacher‐type was then constructed in the schematic window. After parameterizing the length of the transmission lines, it was required to insert the Msub controller, the LSSP controller, and the Harmonic Balance controller, which was used to set the dielectric substrate parameters, the S‐parameter simulation of the circuit's input port, and the Harmonic Balance simulation, respectively. A layout can be constructed for the microstrip lines in the design, and electromagnetic simulation calculations based on the method of moments can be carried out on it to increase the simulation's accuracy. Finally, the lumped components in the schematic design and the S‐parameter results of the microstrip lines which are acquired from the layout simulation were co‐simulated.

### Simulation of Triboelectric Potential Distributions

Finite element simulation software was utilized to simulate the electrical potential of the fabric‐based TENG in the sliding freestanding triboelectric‐layer mode. First, the two‐dimensional spatial modeling approach and the electrostatic mode of AC/DC were selected to simulate the electrical potential of the fabric‐based TENG. Then, one rectangle (54 mm × 100 mm) of FEP material at the top was set as the freestanding triboelectric layer. Third, two rectangles (54 mm × 100 mm) of conductive fabric material with a horizontal spacing of 2 mm at the bottom were set as electrodes. The outermost rectangle was set as the infinite element domain and its interior was given the air material. Afterward, as illustrated in Figure S23 (Supporting Information), the boundary setup for the open‐circuit condition was completed. Finally, the simulation process was conducted to calculate the potential difference between the two electrodes of the fabric‐based TENG and the results were presented in Figure 3b.

### Electrical Characteristic Measurement of Fabric‐Based F‐TENG

To evaluate the electrical output characteristic of the fabric‐based F‐TENG, a commercial linear mechanical reciprocating motor was used to enable the horizontal sliding of the freestanding layer. First, the fabric substrate with two triboelectric electrodes was secured to the experimental table. Then a sheet of FEP film was adhered beneath an acrylic supporting plate, and a weight was fastened on top of the plate. Finally, the weight was fixed to the terminal of the motor's push–pull rod, thus realizing the reciprocating motion of the freestanding triboelectric layer in the horizontal direction. The open‐circuit voltage of the fabric‐based TENG can be measured by connecting the two triboelectric electrodes to an oscilloscope (model: Keysight DSOX3024T). In addition, the short‐circuit current was measured by a multimeter.

## Conflict of Interest

The authors declare no conflict of interest.

## Supporting information

Supporting Information

Supplemental Video 1

## Data Availability

The data that support the findings of this study are available from the corresponding author upon reasonable request.
