# Supplementary material for: Wearable All‐Fabric Hybrid Energy Harvester to Simultaneously Harvest Radiofrequency and Triboelectric Energy
Source: Adv Sci (Weinh). 2024 Feb 21;11(17):2309050. doi: 10.1002/advs.202309050 (PMC11077651; doi:10.1002/advs.202309050)
Supplement: Supplementary file 1 — Supporting Information [file ADVS-11-2309050-s001.pdf]

ADVANCED SCIENCE
Open Access

Supporting Information

for *Adv. Sci.*, DOI 10.1002/advs.202309050

Wearable All-Fabric Hybrid Energy Harvester to Simultaneously Harvest Radiofrequency and Triboelectric Energy

Zhenghao Kou, Chao Zhang, Buyun Yu, Hao Chen, Zhenguo Liu and Weibing Lu**

Supporting Information

Wearable All-fabric Hybrid Energy Harvester to Simultaneously Harvest Radiofrequency and Triboelectric Energy

Zhenghao Kou, Chao Zhang, Buyun Yu, Hao Chen, Zhenguo Liu, Weibing Lu**

Z. H. Kou, C. Zhang, B. Y. Yu, H. Chen, Z. G. Liu, W. B. Lu
State Key Laboratory of Millimeter Waves, School of Information Science and Engineering, Southeast University, Nanjing 210096, P. R. China.
E-mail: liuzhenguo@seu.edu.cn, wblu@seu.edu.cn

Z. H. Kou, C. Zhang, B. Y. Yu, H. Chen, Z. G. Liu, W. B. Lu
Center for Flexible RF Technology, Southeast University, Nanjing 210096, P. R. China.

Z. G. Liu, W. B. Lu
Purple Mountain Laboratories, Nanjing 210096, P. R. China

Keywords:
hybrid energy harvester, rectenna, triboelectric nanogenerator, power management, FCB-SMT

Supporting Notes

Note S1. Detailed derivation between square resistance and conductivity.

When the length of the film sample to be measured is L, the width is W, the thickness is d, and $L = W$, the definition of square resistance is

$$R_{\square} = \frac{\rho \cdot L}{S} = \frac{\rho \cdot L}{W \cdot d} = \frac{\rho}{d} \cdot \frac{L}{W} = \frac{\rho}{d} \qquad \text{(Equation S1)}$$

Based on the square resistance and thickness of the conductive fabric that has been measured, its conductivity can be calculated using the following equation as

$$\kappa = \frac{1}{\rho} = \frac{1}{R_{\square} \cdot d} = \frac{1}{4.409 \times 10^{-3} \left(\Omega/\square\right) \times 9 \times 10^{-5} \left(\mathrm{m}\right)} \approx 2.52 \times 10^{6} \quad \left(\mathrm{S/m}\right)$$

(Equation S2)

Note S2. Theoretical calculation formulas for circular patch antenna cavity modeling.

In the cavity model theory, a circular resonant cavity is assumed to be formed between the circular patch and the ground floor. And the top and bottom surfaces of the cavity are electric walls and the sides of the cavity are magnetic walls. The empirical formula for the resonant frequency of a circular antenna is

$$f_r\left(\mathrm{GHz}\right) = \frac{15K_{mn}}{\pi a_e\left(\mathrm{cm}\right)\sqrt{\varepsilon_r}} \qquad \text{(Equation S3)}$$

Where a_e is the equivalent radius of the circular patch

$$a_e = a\sqrt{1 + \frac{2h}{\pi a \varepsilon_r}\left(\ln\frac{\pi a}{2h} + 1.7726\right)} \qquad \text{(Equation S4)}$$

Where h is the thickness of the substrate, ε_r is the relative permittivity. In addition, K_{mn} is the nth root of the derivative of the mth order Bessel function $J_m{}'\left(ka\right)$. When $m = n = 1$, this corresponds to the main mode of operation of the circular patch antenna, i.e., the TM_{11} mode. In this design, based on the measured $\varepsilon_r = 1.67$, $h = 0.128\,\mathrm{cm}$, and the desired operating frequency of 2.45 GHz, the radius of the circular patch can be calculated as 26.5 mm, that is, the diameter D = 53 mm.

Note S3. Characteristic impedance of microstrip lines.

Based on the height (*H*) of the fabric substrate and the width (*W*) of the microstrip line, as well as the dielectric permittivity (ε_r) of the fabric obtained from experimental tests, the characteristic impedance of the microstrip line can be estimated according to the following approximate equation.

$$Z_0 = \frac{\sqrt{\mu_0/\varepsilon_0}}{\sqrt{\varepsilon_{eff}}\left[1.393+\frac{W}{H}+\frac{2}{3}\ln\left(\frac{W}{H}+1.444\right)\right]} \quad (\Omega) \qquad \text{(Equation S5)}$$

Where

$$\varepsilon_{eff} = \frac{\varepsilon_r+1}{2}+\frac{\varepsilon_r-1}{2}\left(1+12\frac{H}{W}\right)^{-1/2} \qquad \text{(Equation S6)}$$

In addition, if the desired characteristic impedance (Z_0) and relative permittivity (ε_r) of the microstrip line are known, the width (*W*) of the microstrip line can be deduced using the following equation.

$$W = \frac{2H}{\pi}\left\{B-1-\ln\left(2B-1\right)+\frac{\varepsilon_r-1}{2\varepsilon_r}\left[\ln\left(B-1\right)+0.39-\frac{0.61}{\varepsilon_r}\right]\right\}$$

(Equation S7)

Where

$$B = \frac{377\pi}{2Z_0\sqrt{\varepsilon_r}} \qquad \text{(Equation S8)}$$

Note S4. Theoretical basis of F-TENG.

From the electrodynamic point of view, the governing equation for all four modes of TENG is the relations of $V-Q-x$, which are expressed in the following equation.

$$V=-\frac{Q}{C}+V_{OC} \qquad \text{(Equation S9)}$$

Where V is the voltage between the two electrodes, V_{OC} is the voltage between two electrodes under open-circuit conditions, C is the total capacitance, and Q is the amount of charge transferred between the two electrodes. While the average transferred charges ($\bar{Q}$) under short-circuit conditions and the instantaneous output power (P) of F-TENG can be given by the following equations.

$$\bar{Q}=4f\frac{S\sigma x_{\max}}{d_0+g} \qquad \text{(Equation S10)}$$

Where $x=A_0+A_0\sin(\omega t)$.

$$P=\left[\frac{2\sigma A_0}{\varepsilon_0}\frac{\omega\varepsilon_0 S(d_0+g)}{(d_0+g)^2+\omega^2\varepsilon_0^2S^2C^2}\left(\omega R\frac{\varepsilon_0 S}{d_0+g}\sin(\omega t)+\cos(\omega t)\right)\right]^2 R$$

(Equation S11)

The time-averaged output power ($\bar{P}$) can be given below

$$\bar{P}=f\int_0^{1/f}Pdt \qquad \text{(Equation S12)}$$

Note S5. PMC parameter setting equations.

(1) MPPT function

The boost input regulation reference is the open circuit voltage at the VIN(ADP) pin scaled to a ratio programmed by the resistor divider (R_{OC1} and R_{OC2}) at the MPPT pin according to the following equation.

$$V_{MPPT} = V_{IN}\left(Open\ Circuit\right)\left(\frac{R_{OC1}}{R_{OC1}+R_{OC2}}\right) \qquad \text{(Equation S13)}$$

Where V_{IN} (Open Circuit) is the input open circuit voltage.

(2) Power Good function

The programmable PGOOD voltage threshold indicates that the SYS voltage is at an acceptable level. It must be set by using external resistors (R_{PG1} and R_{PG2}). The V_{SETPG} falling threshold voltage is given by the following equation.

$$V_{SETPG\_FALLING} = V_{INT\_REF}\left(1+\frac{R_{PG1}}{R_{PG2}+R_{PG\_HYST}}\right) \qquad \text{(Equation S14)}$$

The SETHYST pin can program the hysteresis with an external resistor (R_{PG_HYST}) given by the following equation.

$$V_{SETPG\_RISING} = V_{INT\_REF}\left(1+\frac{R_{PG1}+R_{PG\_HYST}}{R_{PG2}}\right) \qquad \text{(Equation S15)}$$

Where the typical value of the internal reference (V_{INT_REF}) voltage is 1.011V.

(3) Battery overcharging protecting (TERM) function

To prevent rechargeable batteries from being overcharged and damaged, the battery terminal charging threshold (V_{BAT_TERM}) must be set by using external resistors (R_{TERM1} and R_{TERM2}) given by the following equation.

$$V_{BAT\_TERM} = \frac{3}{2}\times V_{INT\_REF}\times\left(1+\frac{R_{TERM1}}{R_{TERM2}}\right) \qquad \text{(Equation S16)}$$

(4) Backup function

When there is a backup power supply in the circuit, it is optional to turn on the BACK_UP function and set the switching threshold on the BAT pin by using external resistors (R_{BK1} and R_{BK2}) at the SETBK pin.

$$V_{SETKB} = V_{INT\_REF}\left(1+\frac{R_{BK1}}{R_{BK2}}\right) \qquad \text{(Equation S17)}$$

(5) Battery stop discharging protecting (SD) function

The PMC designed in this paper has two discharging modes, one is directly connecting the storage element to the load for driving and the other is releasing the energy at the REG_OUT pin. For the second discharging mode, the stop discharging threshold (V_{SETSD}) of the battery can be set by the following equation to protect it from over-discharging and damage.

$$V_{SETSD} = V_{INT\_REF}\left(1+\frac{R_{SD1}}{R_{SD2}}\right) \qquad \text{(Equation S18)}$$

Supporting Figures

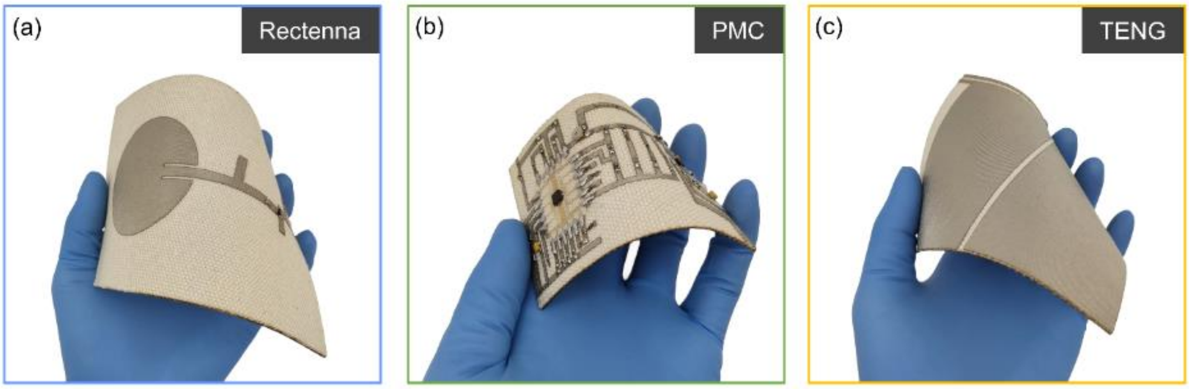

Figure S1. Photo images of the fabricated regular fabric modules: (a) all-fabric rectenna; (b) fabric-based PMC; (c) all-fabric TENG.

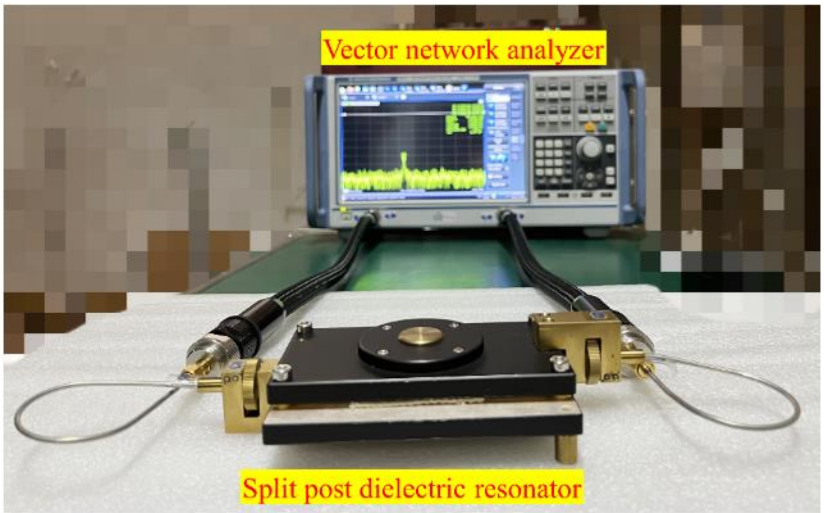

Figure S2. Photo image of electromagnetic characteristic measurement of the cotton canvas.

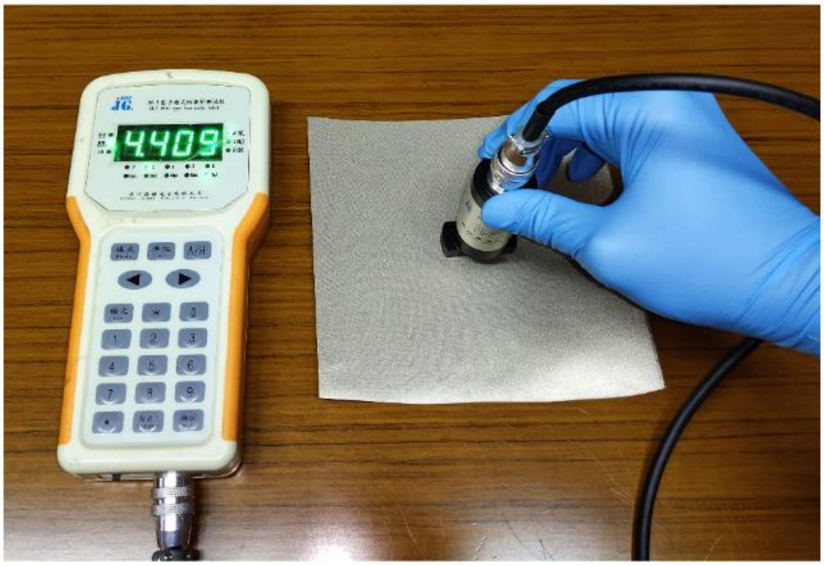

Figure S3. Photo image of square resistance measurement of the conductive fabric.

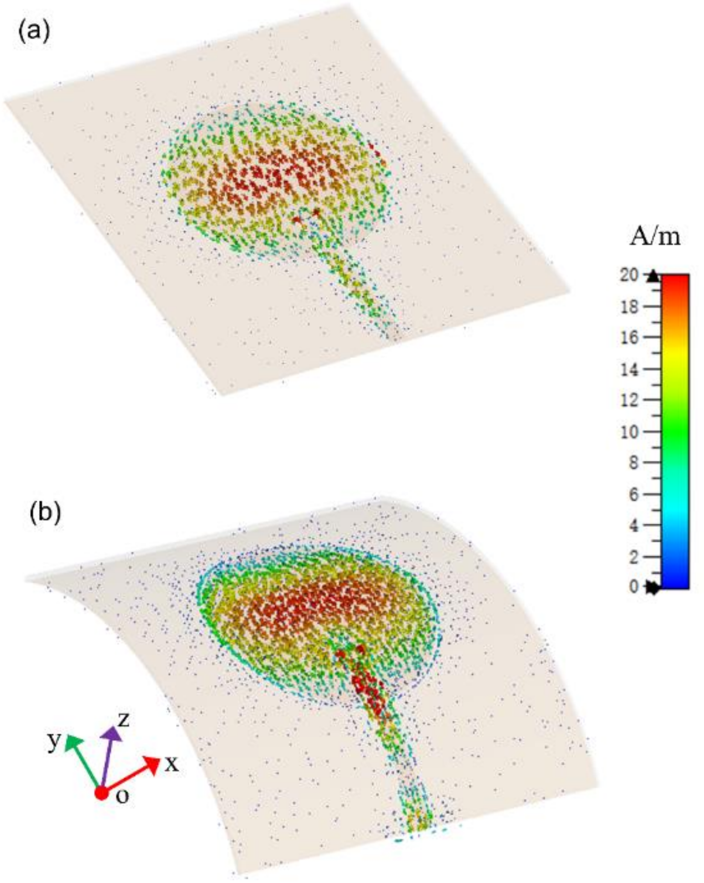

Figure S4. Simulated surface current distributions of the patch antenna at 2.45 GHz when it is (a) flat and (b) bent.

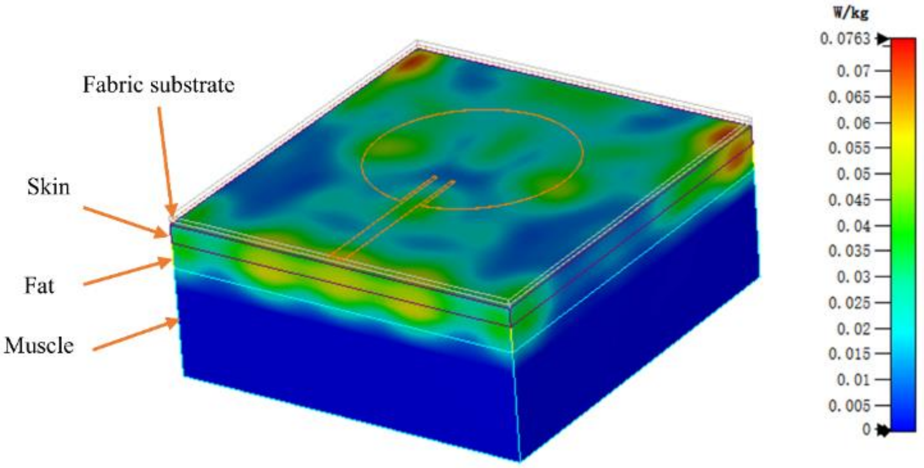

Figure S5. Simulated SAR distributions of the all-fabric antenna on the phantom when 1 W of power is used.

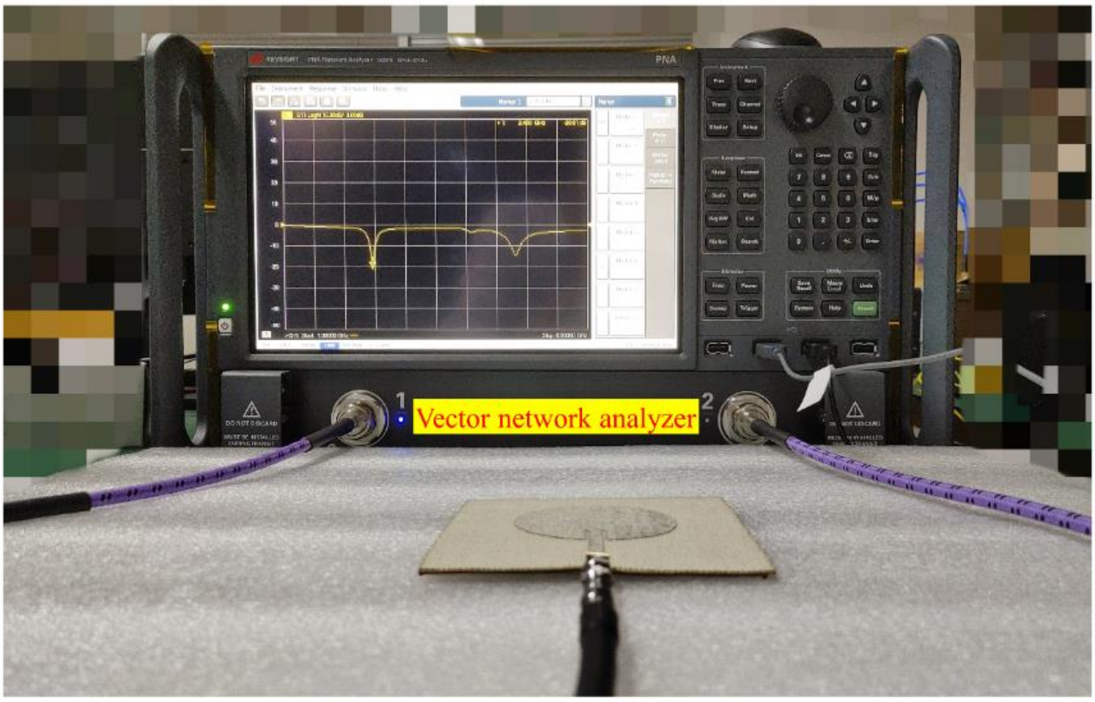

Figure S6. Photo image of S-parameters measurement of the all-fabric patch antenna when flat.

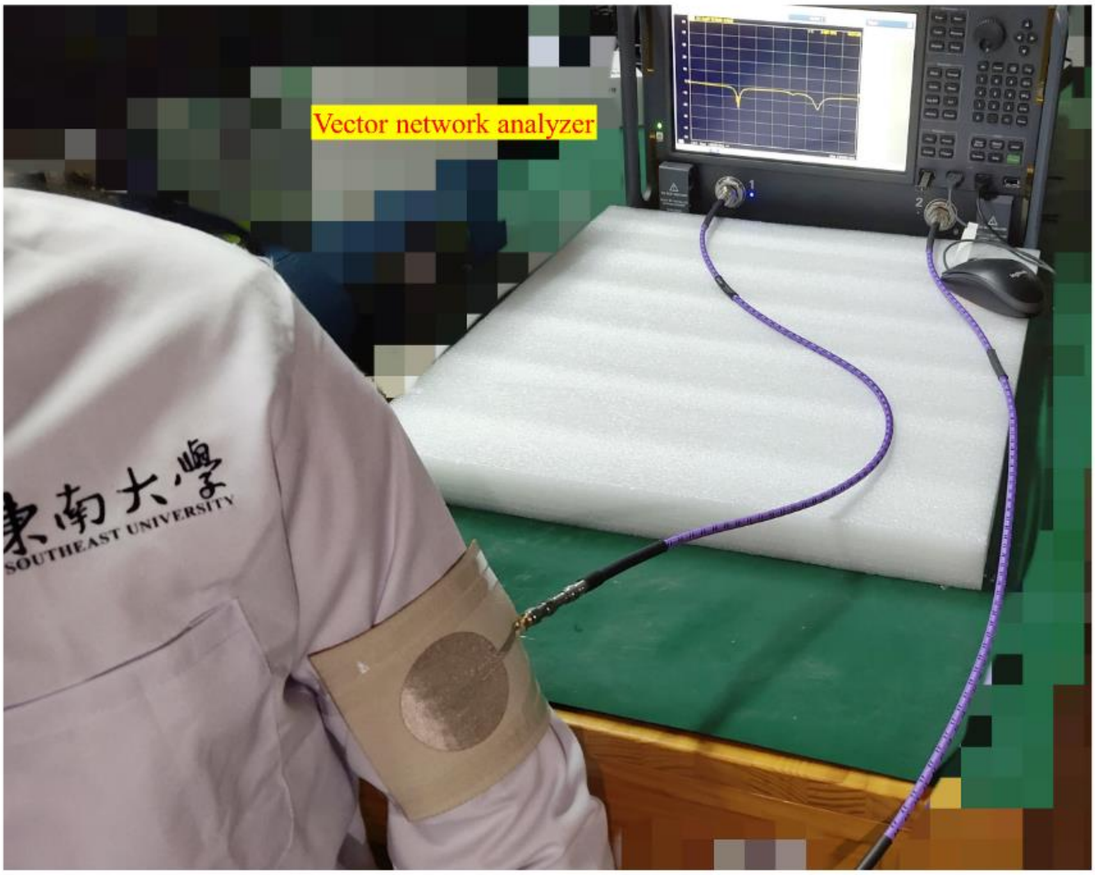

Figure S7. Photo image of S-parameters measurement of the all-fabric patch antenna when it is attached on the human arm.

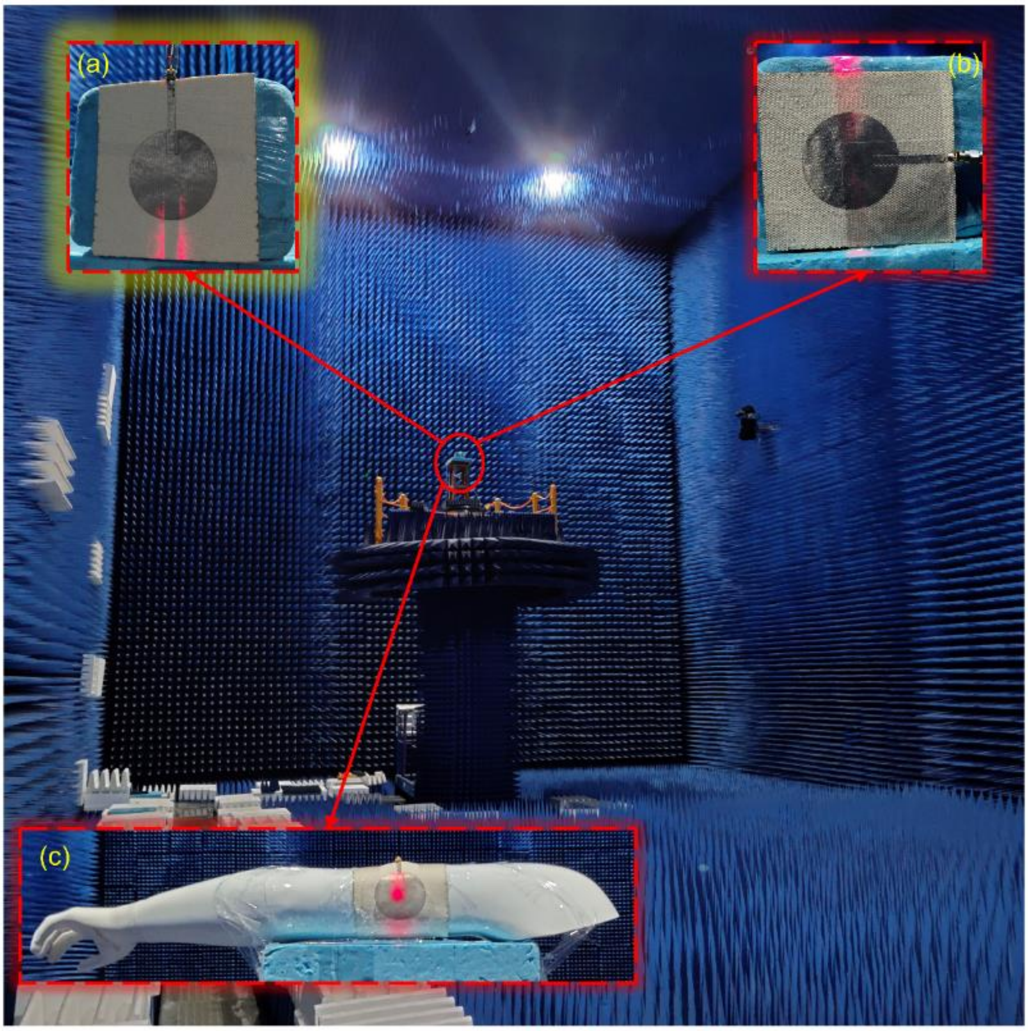

Figure S8. Photo images of the radiation characteristic measurements of the all-fabric: (a) *xoz* plane when flat; (b) *yoz* plane when flat; (c) *xoz* plane bending on human arm model. The red laser on the sample serves as the reference position where the DUT needs to be mounted.

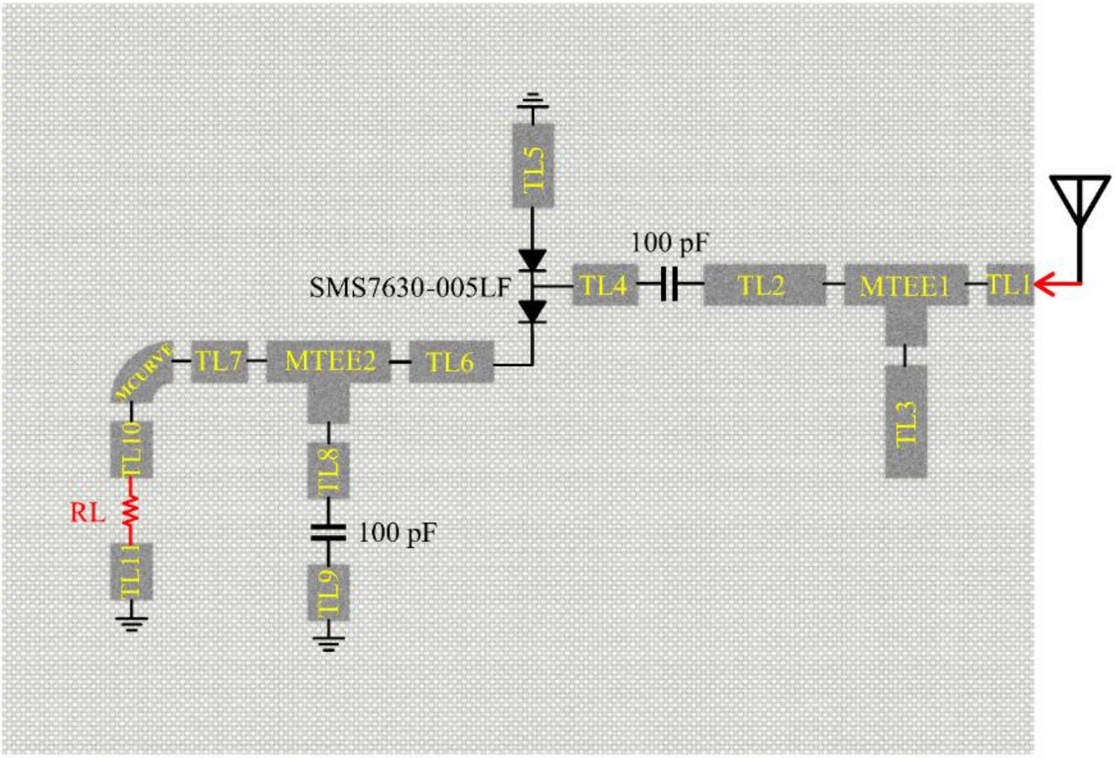

Figure S9. Schematic diagram of the fabric-based RF rectifying circuit.

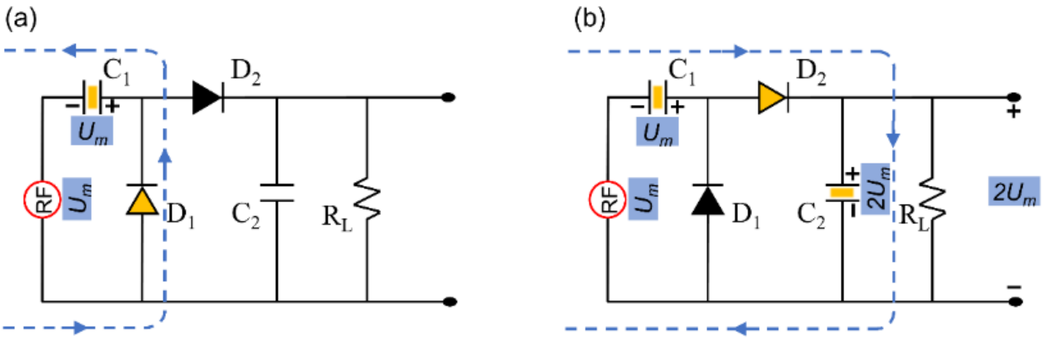

Figure S10. Schematic diagram of the doubler voltage rectifying circuit, depicting (a) C_1 charging stage and (b) C_2 charging stage.

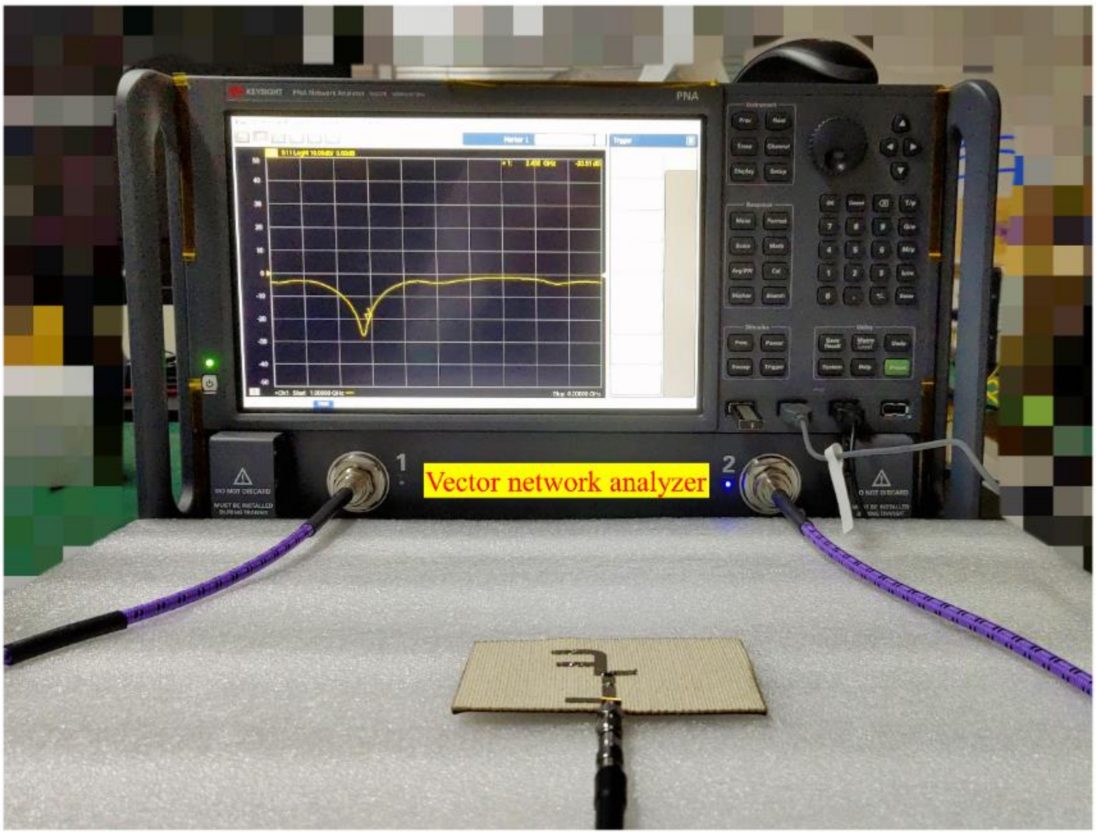

Figure S11. Photo image of S-parameters measurement of the fabric-based rectifying circuit when it is flat.

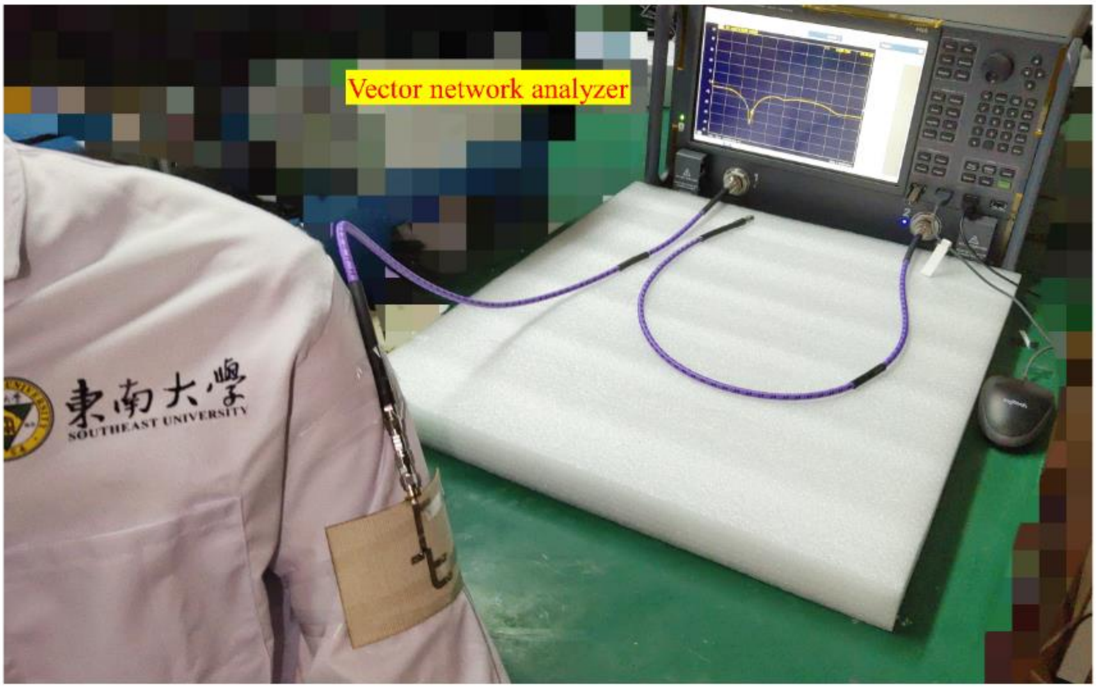

Figure S12. Photo image of S-parameters measurement of the fabric-based rectifying circuit when it is attached on the human arm.

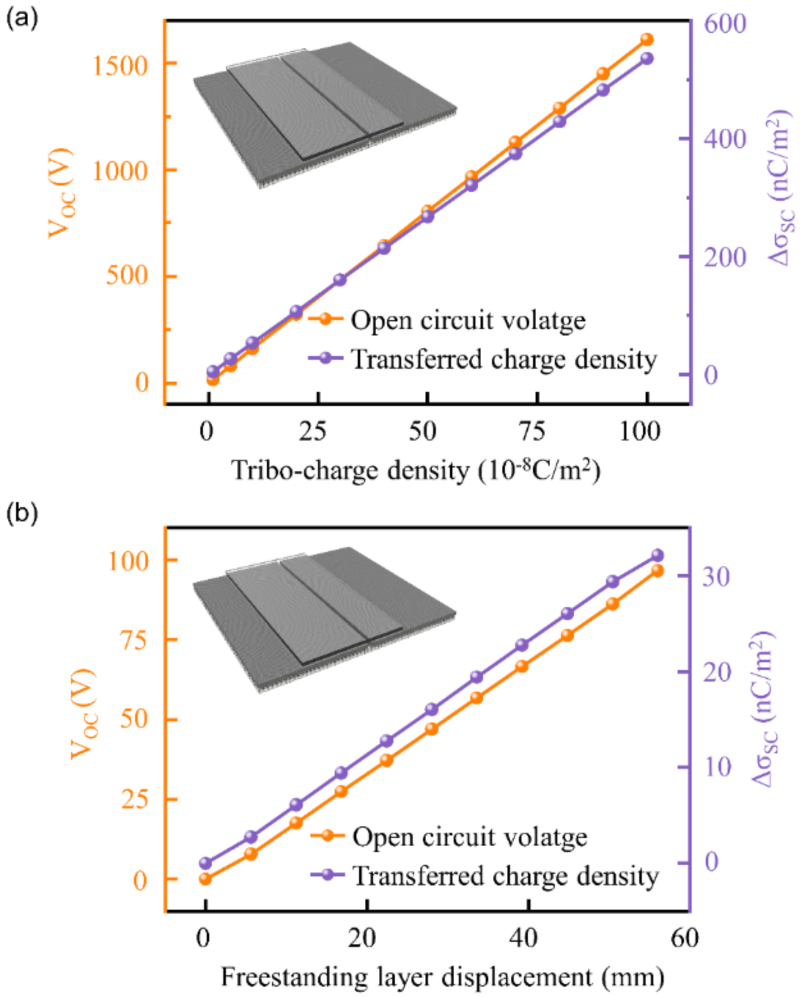

Figure S13. The simulated potential distributions under open circuit conditions and transferred charge density under short circuit conditions: (a) at different assumed tribo-charge densities; and (b) at different sliding displacements.

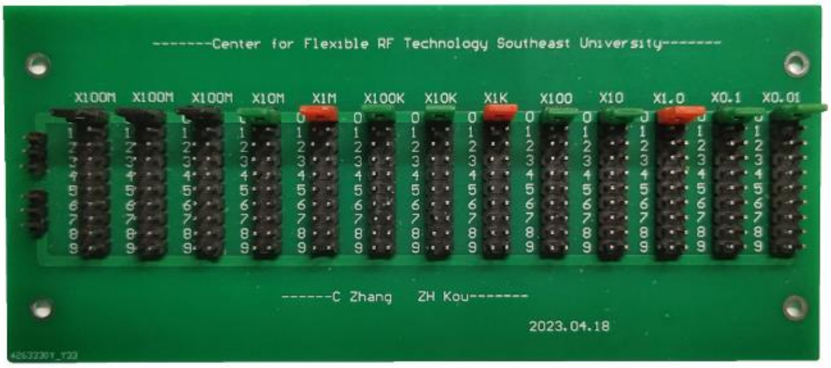

Figure S14. Photo image of the self-designed 11-bit programmable resistor.

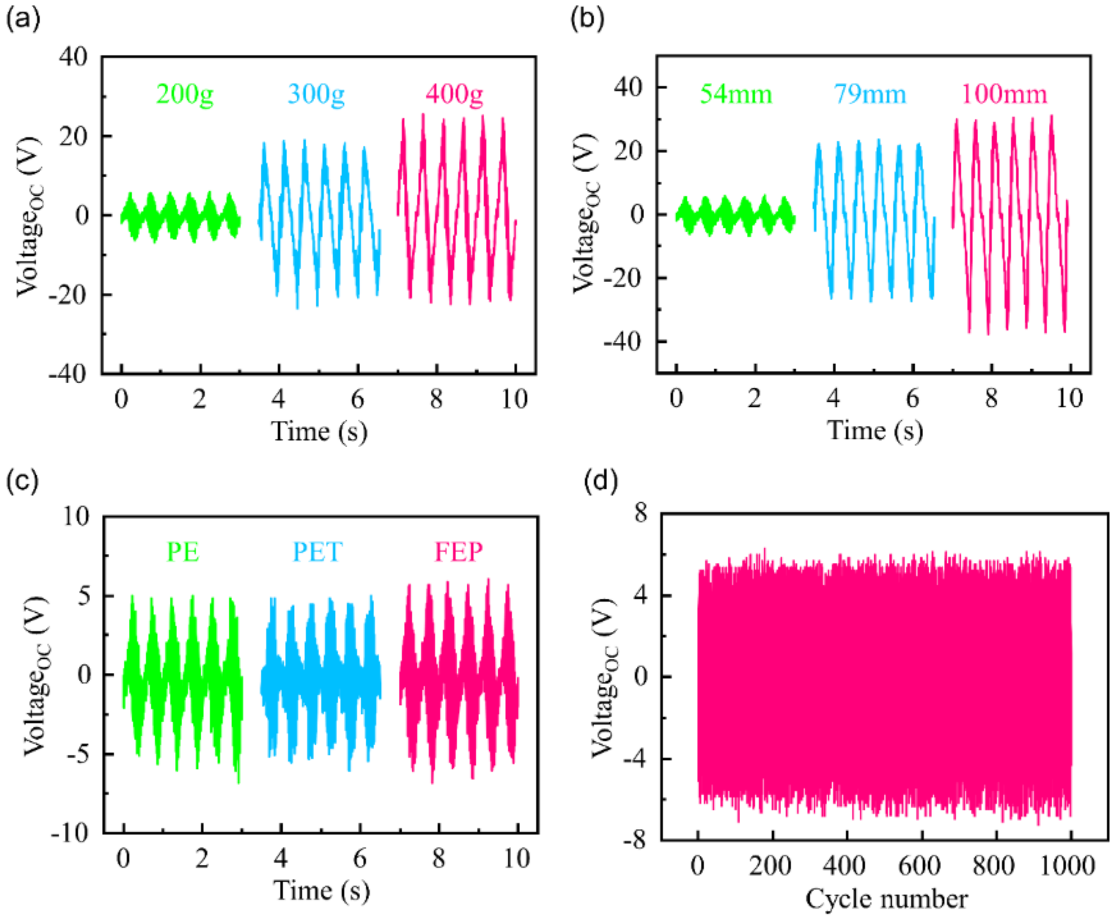

Figure S15. Evaluation of TENG performance concerning applied force, contact areas, freestanding triboelectric layer materials, and durability. (a) Forces are applied with weights of 200 g, 300 g, and 400 g, respectively. (b) Under a fixed width of 100 mm, the contact lengths are 54 mm, 79 mm, and 100 mm, respectively. (c) PE, PET, and FEP are used as freestanding triboelectric layer materials, respectively. (d) Open circuit voltage of TENG during 1000 cycles at a frequency of 2 Hz.

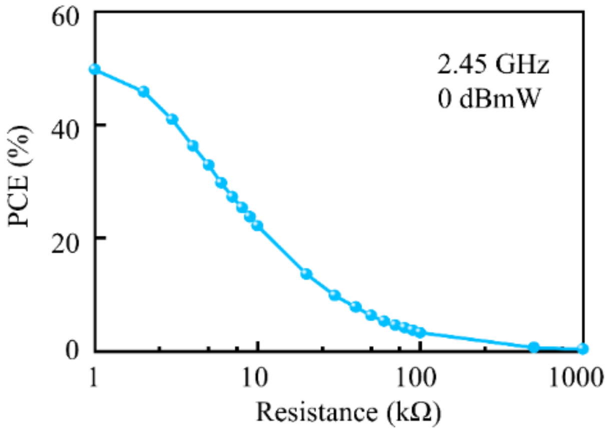

Figure S16. Dependence of the RF-to-DC power conversion efficiency of rectifying circuit on different load resistances.

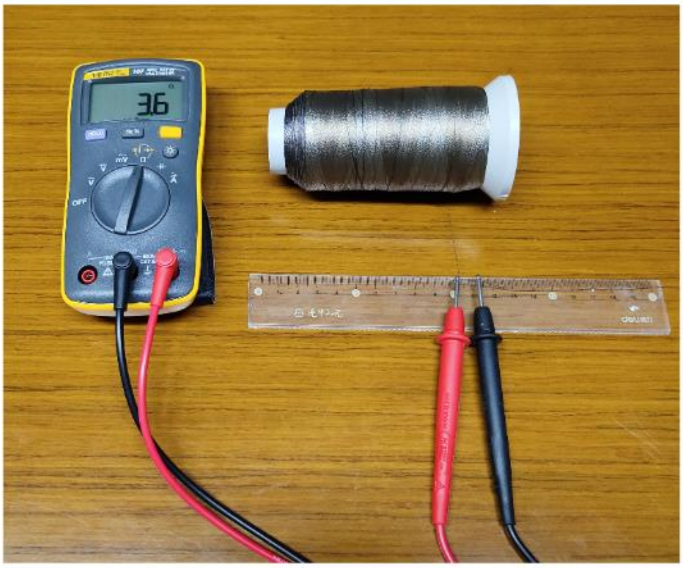

Figure S17. Photo image of resistance characteristic measurement of the conductive thread.

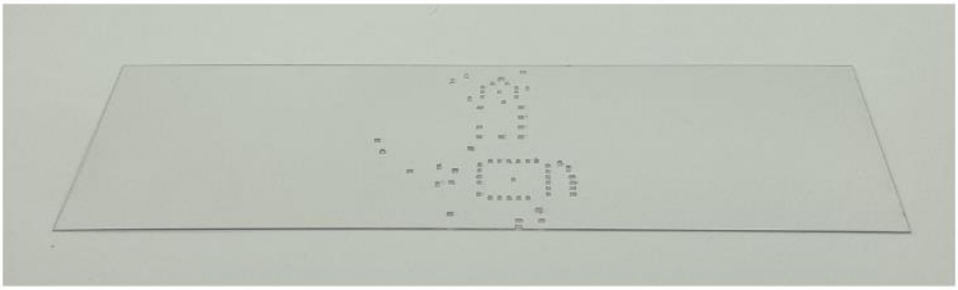

Figure S18. Photo image of a hollowed acrylic plate for applying solder paste.

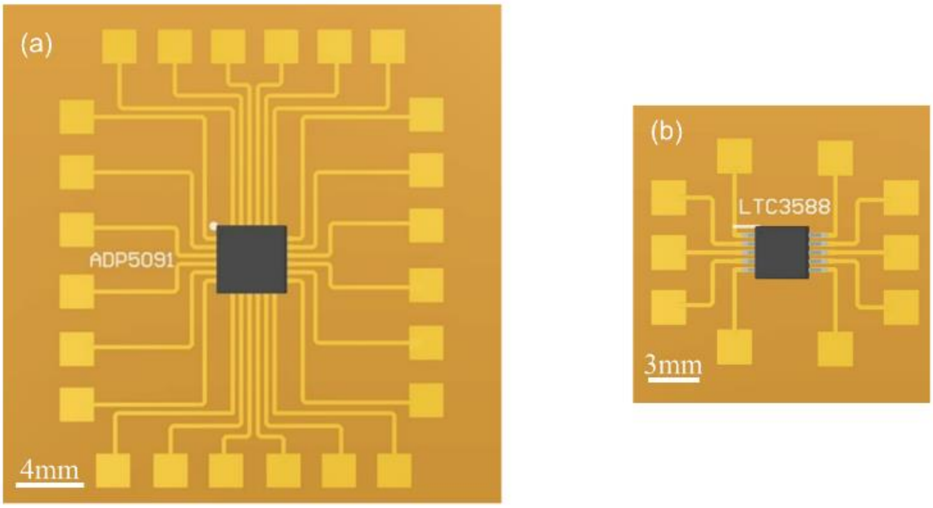

Figure S19. Schematic diagram of “pins expansion” circuit board based on PET substrate: (a) ADP5091 chip; (b) LTC3588 chip.

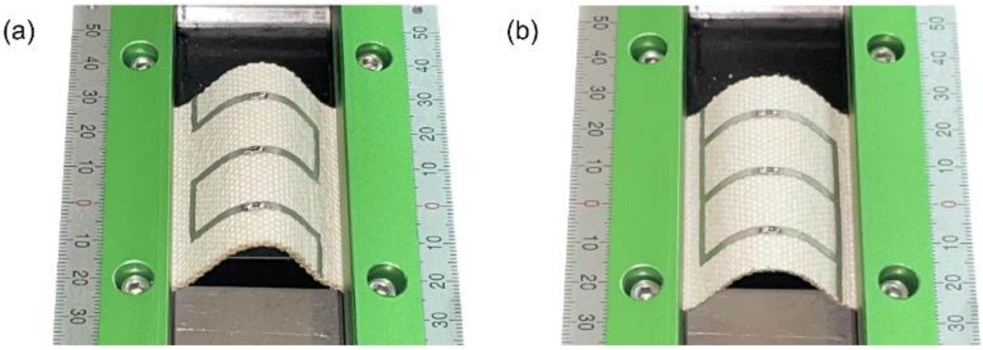

Figure S20. Bending fatigue test photo images of the circuit with (a) series and (b) parallel configurations. (model: Nanoupe FlexTest-S-P2)

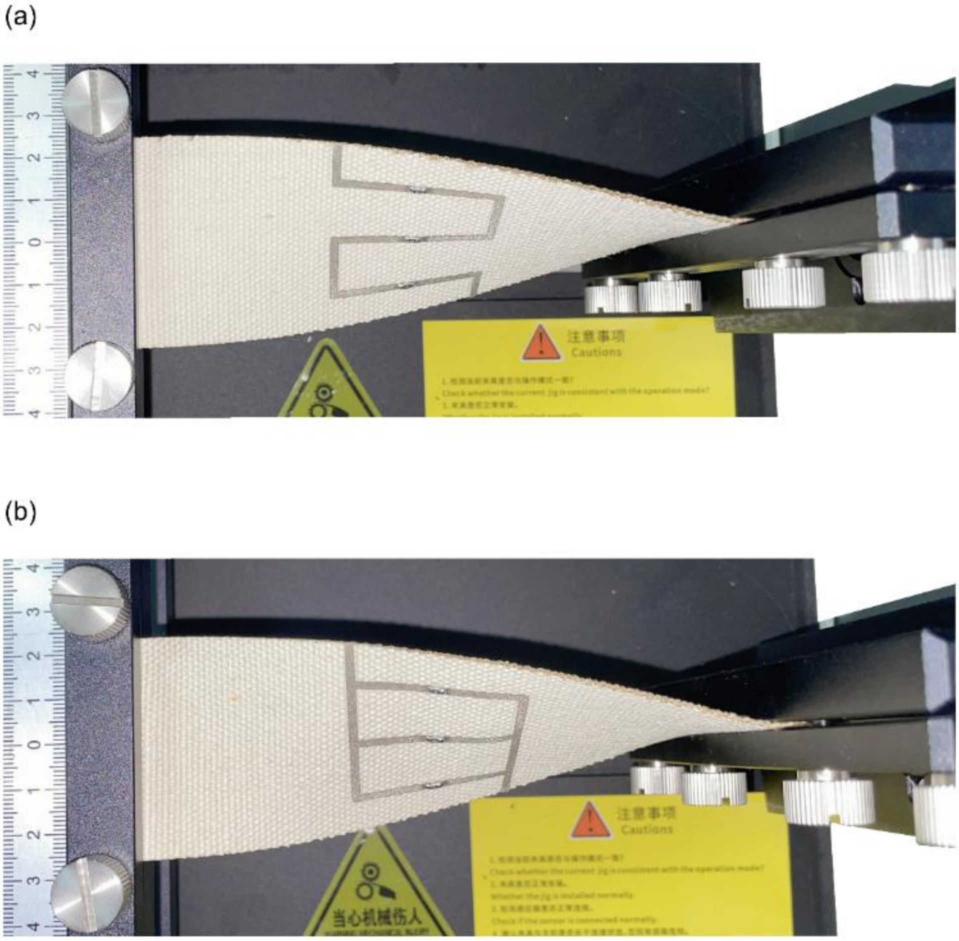

Figure S21. Torsional fatigue test photo images of the circuit with (a) series and (b) parallel configurations. (model: Nanoupe FlexTest-T-F)

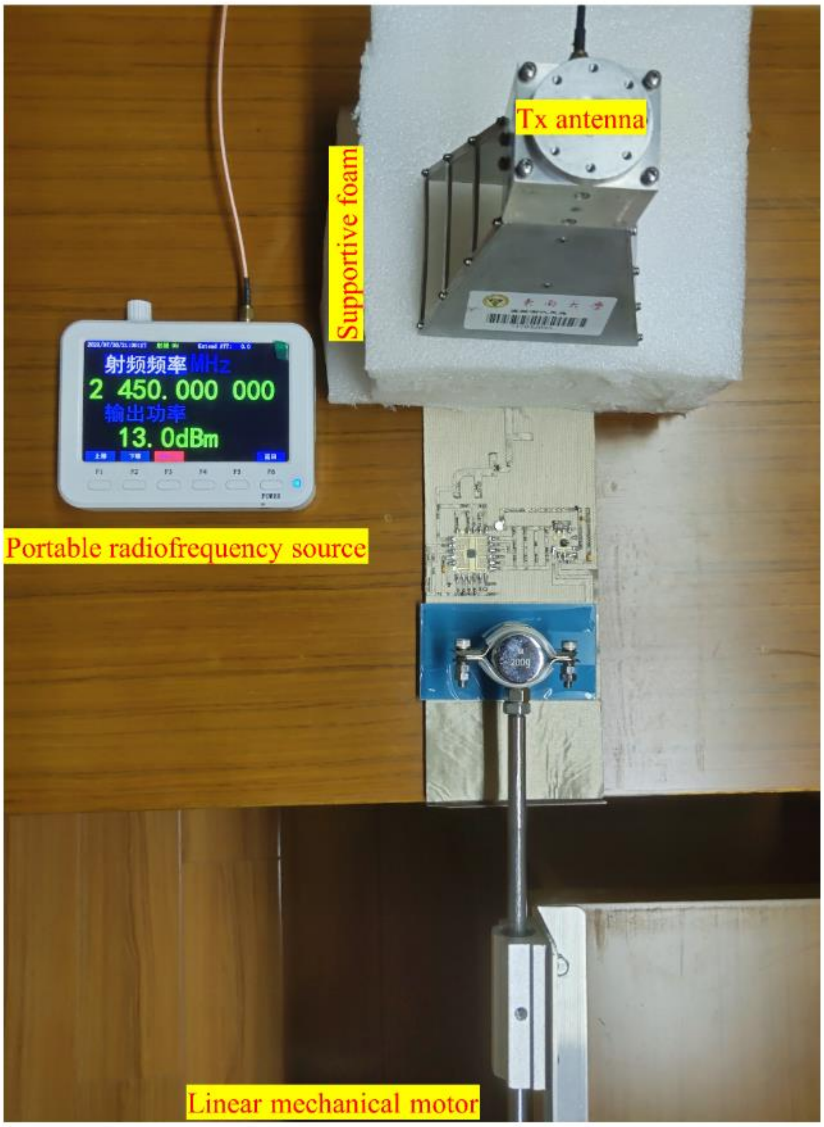

Figure S22. Image of the RF energy harvesting and TE energy harvesting test environment setups.

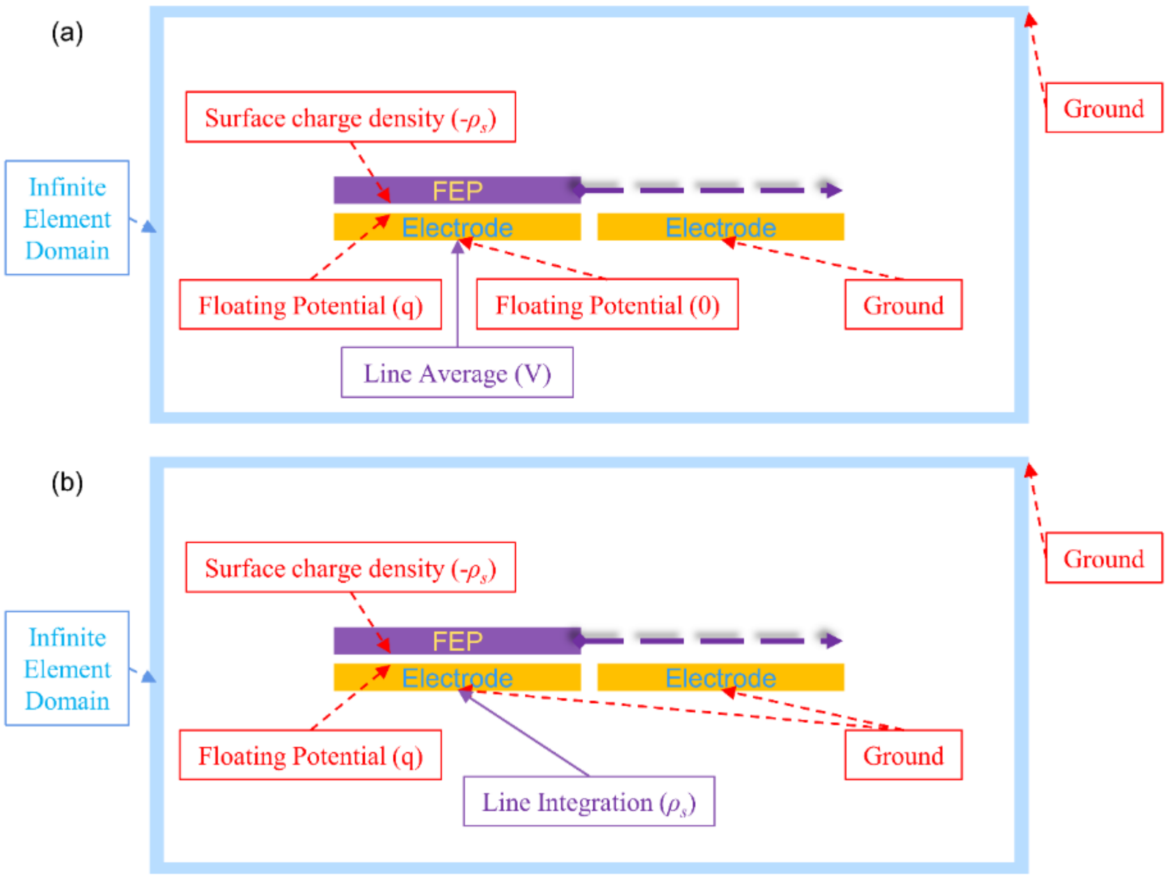

Figure S23. Schematic of boundary condition settings for F-TENG simulation: (a) open circuit conditions; (b) short circuit conditions.

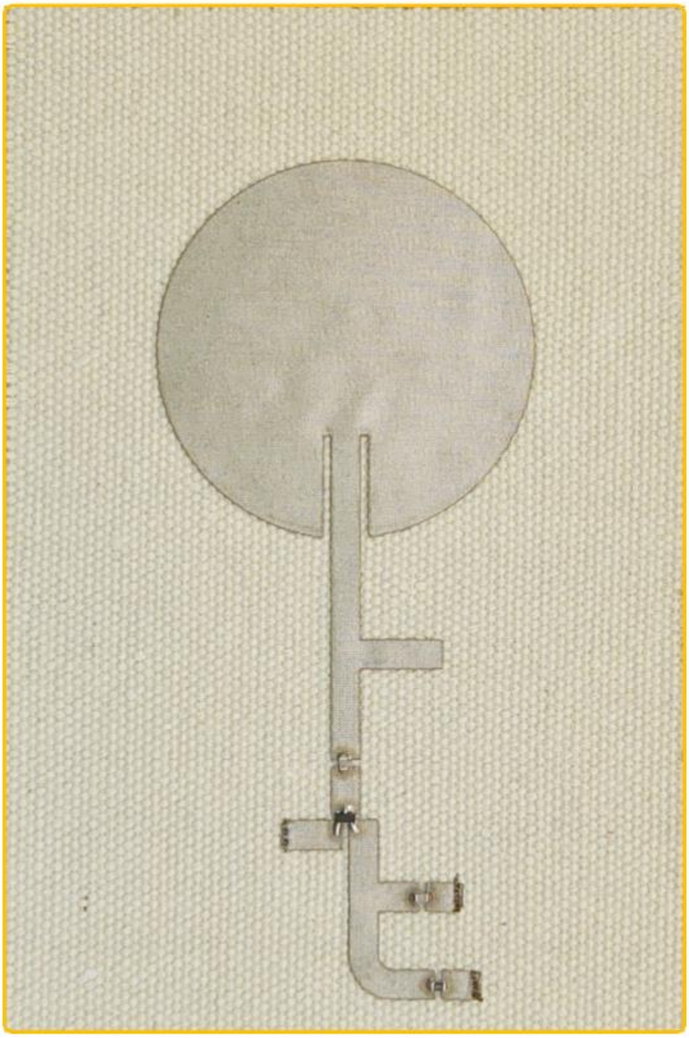

Figure S24. Top view of the prepared fabric-based RF rectifying circuit sample.

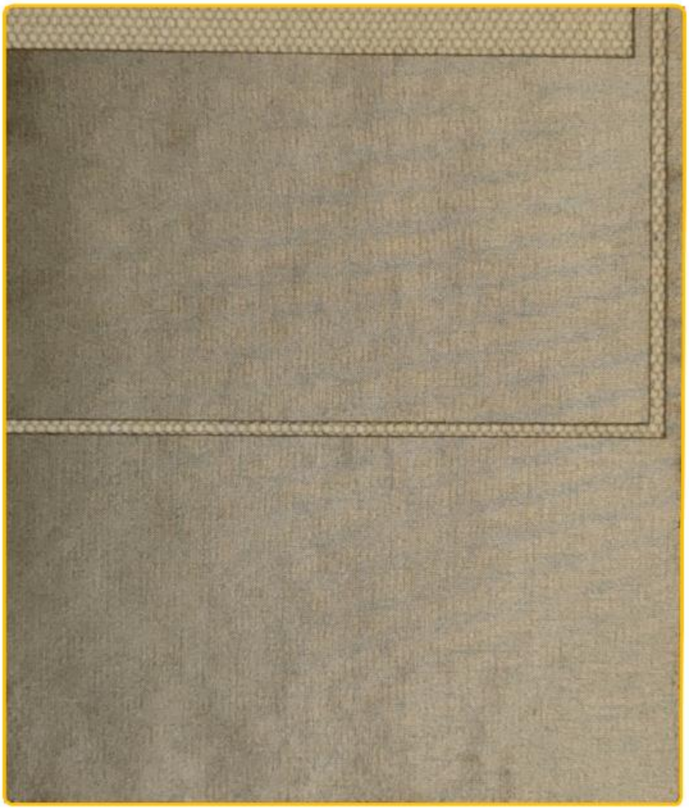

Figure S25. Top view of the prepared all-fabric TENG sample.

(a)

(b)

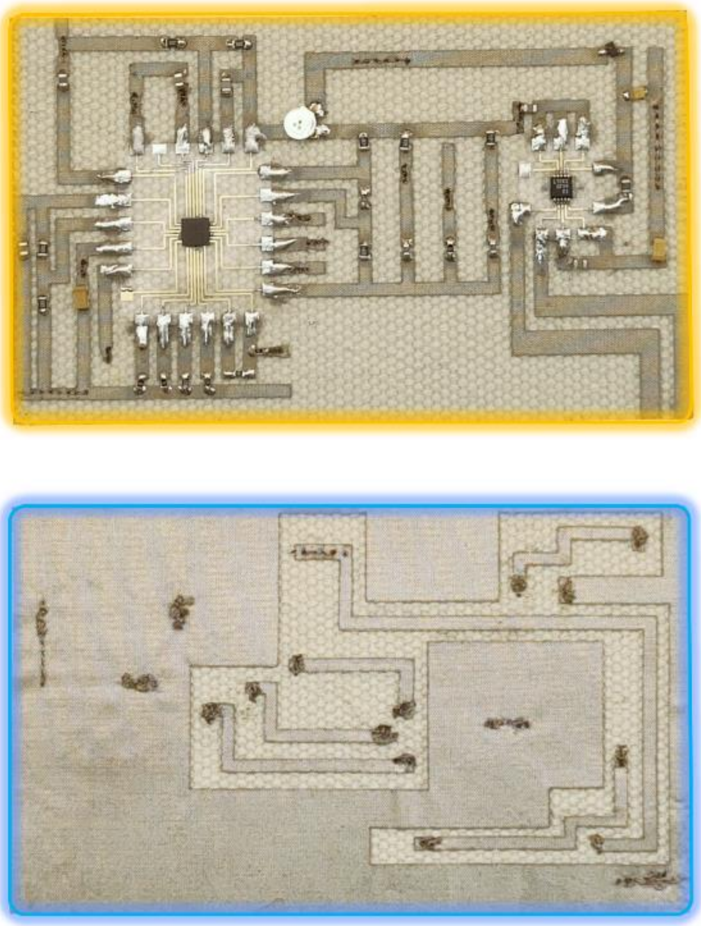

Figure S26. (a) Top view and (b) bottom view of the assembled fabric-based PMC sample.

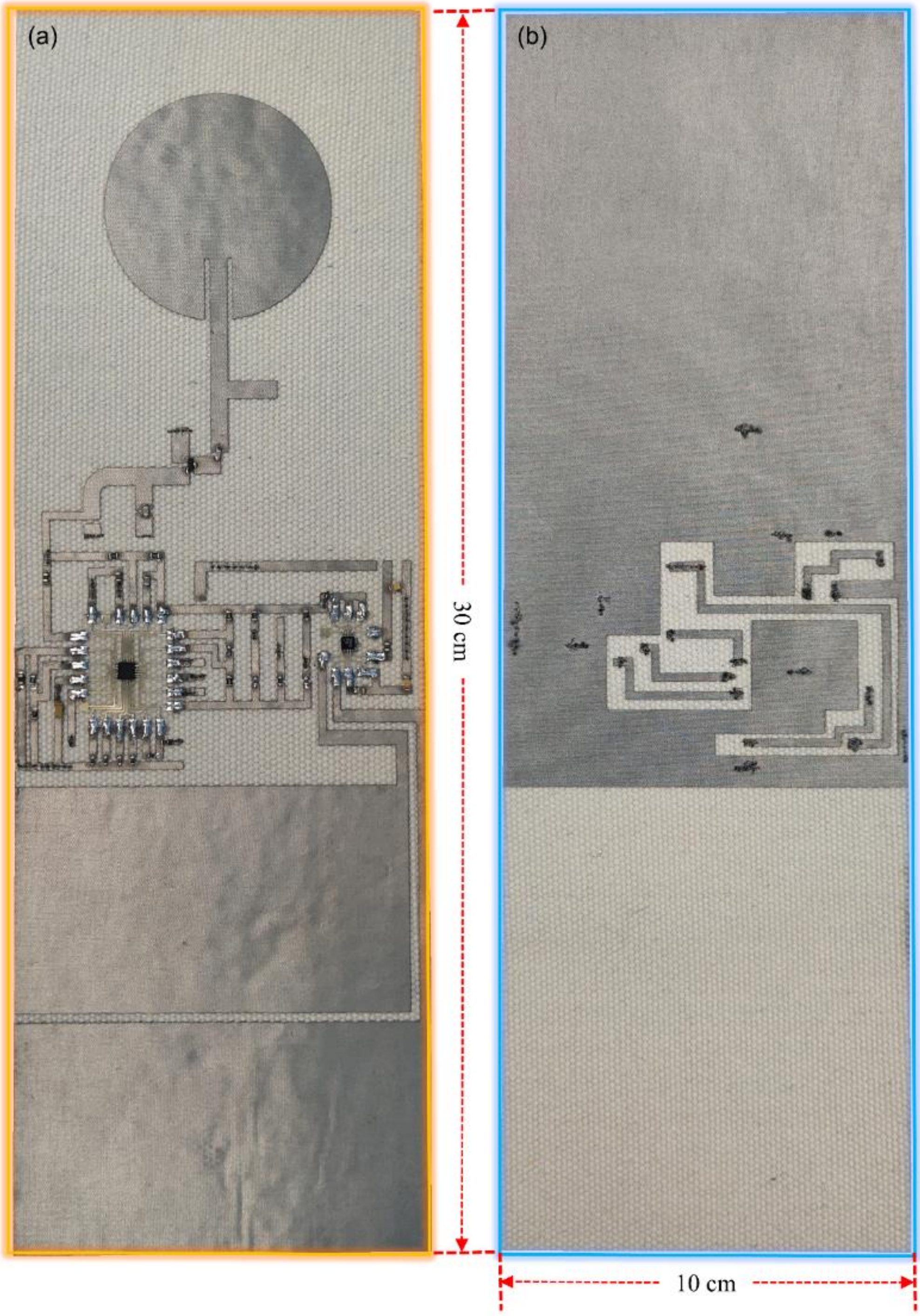

Figure S27. (a) Top view and (b) bottom view of the fabricated fabric-based RF-TE hybrid energy harvesting system.

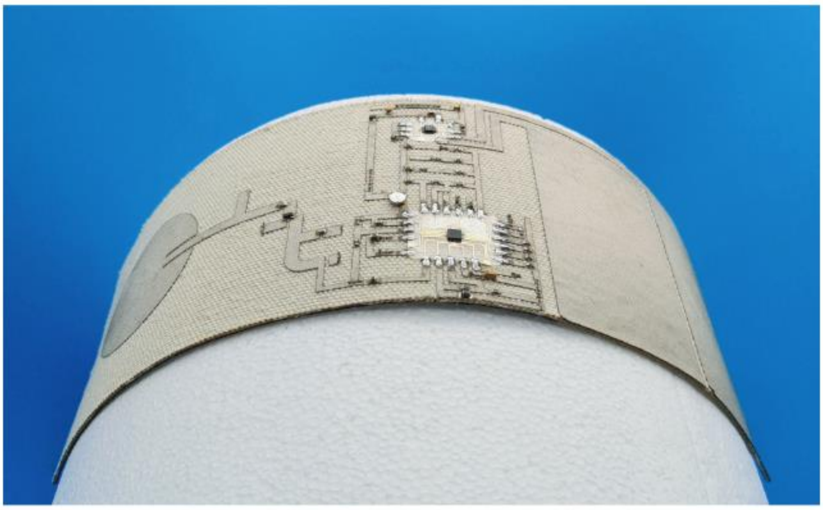

Figure S28. Photograph of the fabric-based RF-TE hybrid energy harvester sample in its bending shape.

Supporting Tables

Table S1. The structural parameters of the RF rectifying circuit.

Transmission line	Length (unit: mm)
TL1	5
TL2	13
TL3	12
TL4	7
TL5	9
TL6	9
TL7	6
TL8	6
TL9	6
TL10	6
TL11	6
MCURVE	Radius: 4.4
All	Width: 4.4

Table S2. Configuration of lumped components of the PMC.

Lumped component	Value
L_1	22 μH
L_2	10 μH
C_1	4.7 μF
C_2	10 nF
C_3	3.3 μF
C_4	4.7 μF
C_5	1 μF
C_6	4.7 μF
C_7	1 μF
C_8	1 μF
R_{OC1}	10 MΩ
R_{OC2}	10 MΩ
R_{OUT1}	10 MΩ
R_{OUT2}	10 MΩ
R_{OP}	1 kΩ
R_{SD1}	5.9 MΩ
R_{SD2}	4.12 MΩ
R_{PG1}	6.19 MΩ
R_{PG2}	3.83 MΩ
R_{PG_HYST}	100 kΩ
R_{BK1}	0 Ω
R_{BK2}	OPEN
R_{TERM1}	5.9 MΩ
R_{TERM2}	4.12 MΩ
D_1	HSMS-286c

Supporting Video

Video S1. Application of the RF-TE HEH to harvest the wireless energy and biomechanical energy to sustainably power a mechanical watch.
